# Supplementary material for: Income and racial disparity in household publicly available electric vehicle infrastructure accessibility
Source: Nat Commun. 2024 Jun 14;15:5106. doi: 10.1038/s41467-024-49481-w (PMC11178907; doi:10.1038/s41467-024-49481-w)
Supplement: Supplementary file 1 — Supplementary Information [file 41467_2024_49481_MOESM1_ESM.pdf]

Supplementary Information for

Income and Racial Disparity in Household Publicly Available Electric Vehicle Infrastructure Accessibility

Jiehong Lou[1], Xingchi Shen[2], Deb A. Niemeier[3], Nathan Hultman[1]

[1] Center for Global Sustainability, School of Public Policy, University of Maryland
[2] School of the Environment, Yale University
[3] Dept. of Civil and Environmental Engineering, University of Maryland

This document includes:
Supplementary Figure 1-21, page 2 - page 28
Supplementary Tables 1-7, page 29 - page 39
Supplementary Note 1, Page 40 - page 47
Supplementary Note 2, Page 48 - page 49
Supplementary Note 3, EV Charging Regulations and Policies, Page 50 - page 54
Supplementary Note 4, EV A systematic literature review for EV accessibility and its contributing factors, Page 55 - page 57
Supplementary Note 5, Adopted Terms to Describe Our Research Groups, page 58
Supplementary Reference, Page 59 – page 64

Supplementary information

a

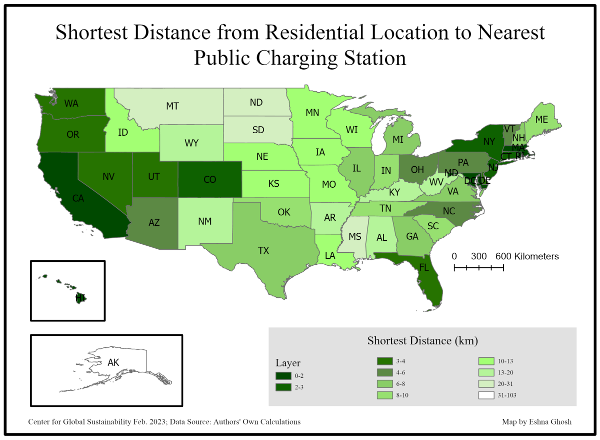

b

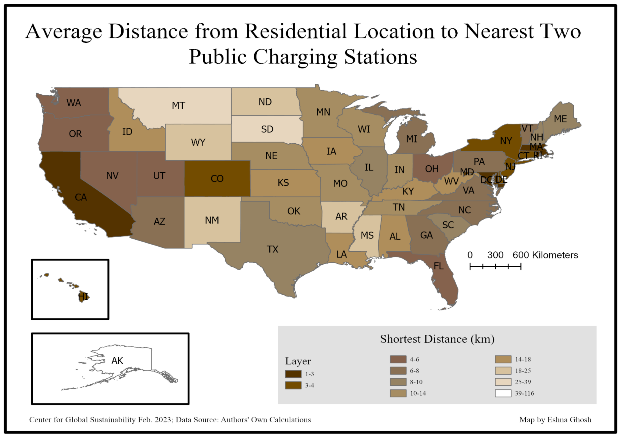

c

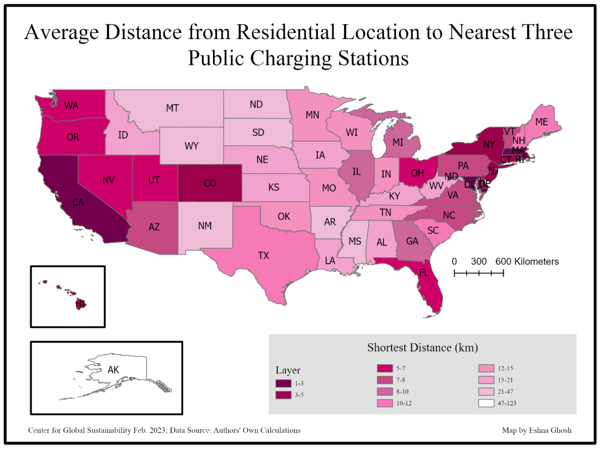

d

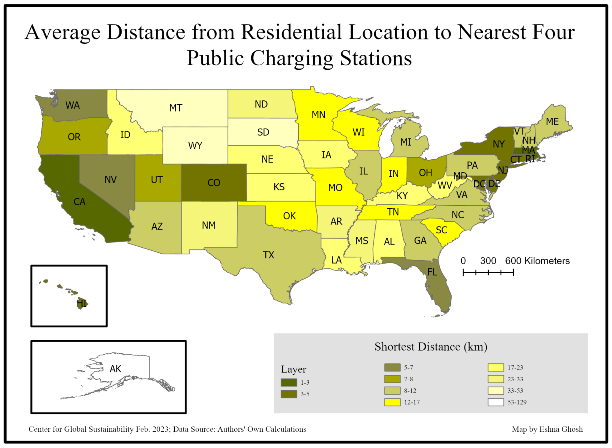

e

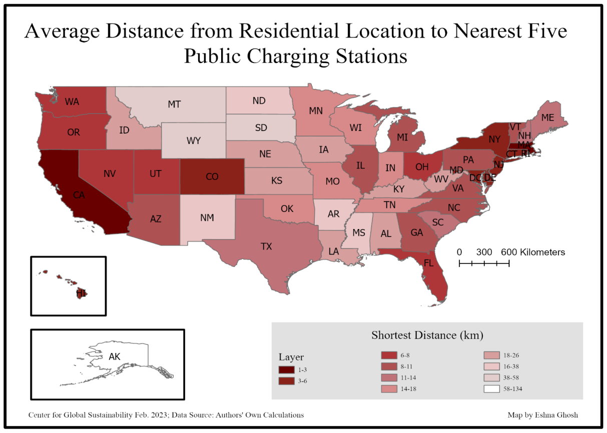

f

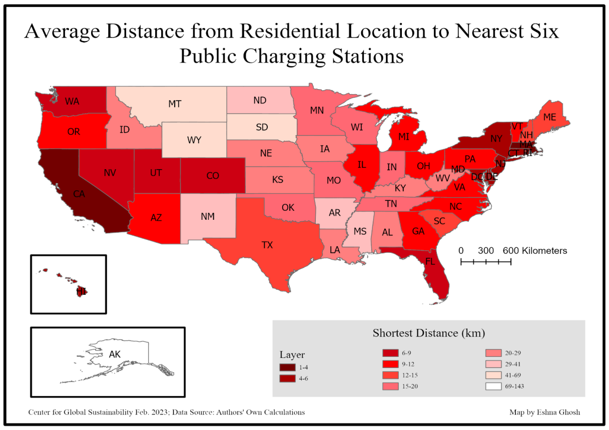

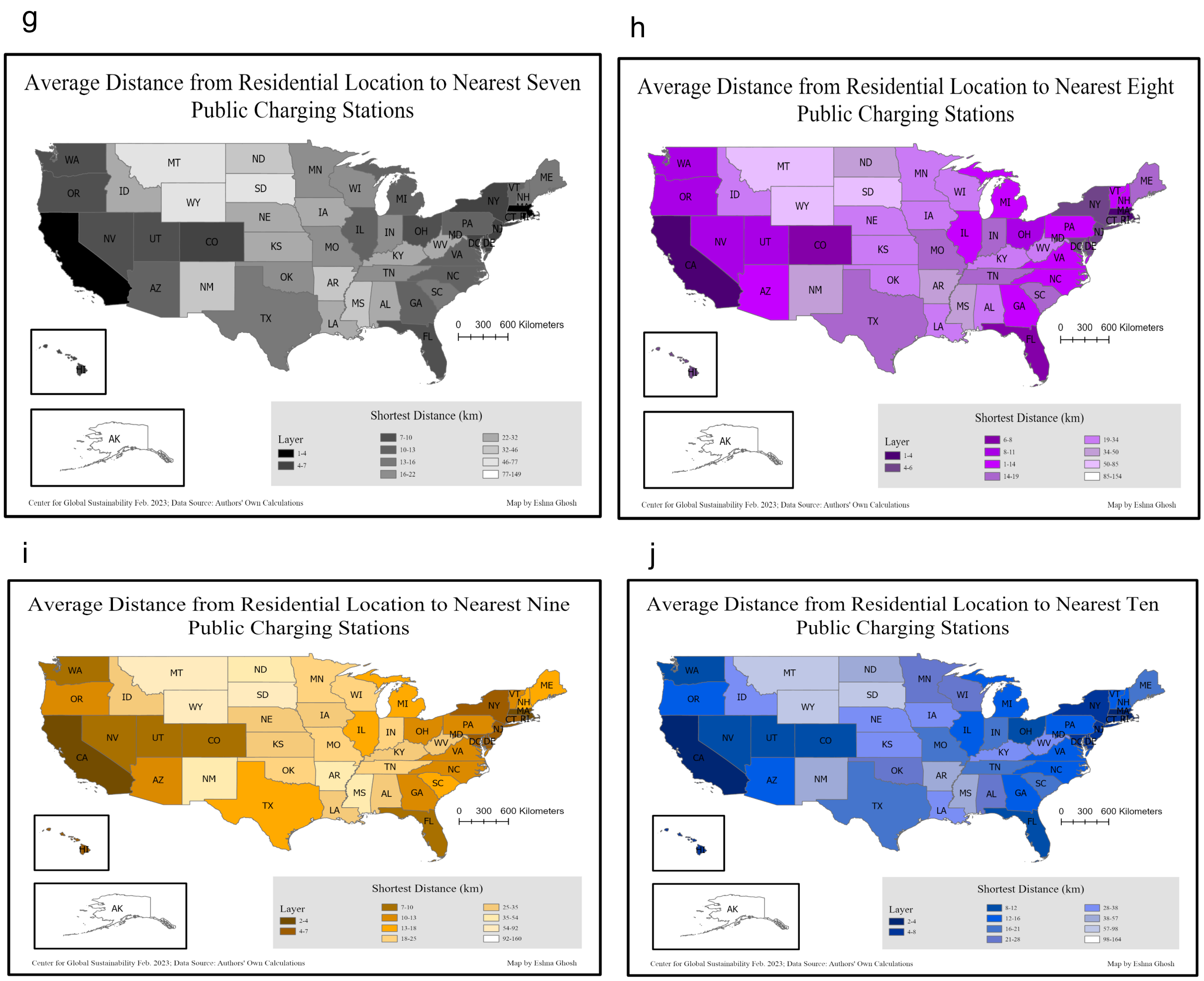

Supplementary Figure 1. Public electric vehicle (EV) infrastructure accessibility for U.S. households. a. Average distance of all the households to the nearest EV public charging station by state (dp1); b. Average distance of all the households to the nearest two public charging stations (dp2); c. Average distance of all the households to the nearest three public charging stations (dp3); d. Average distance of all the households to the nearest four public charging stations (dp4); e. Average distance of all the households to the nearest five public charging stations (dp5); f. Average distance of all the households to the nearest six public charging stations (dp6); g. Average distance of all the households to the nearest seven public charging stations (dp7); h. Average distance of all the households to the nearest eight public charging stations (dp8); i. Average distance of all the households to the nearest nine public charging stations (dp9); j. Average distance of all the households to the nearest 10 public charging stations (dp10).

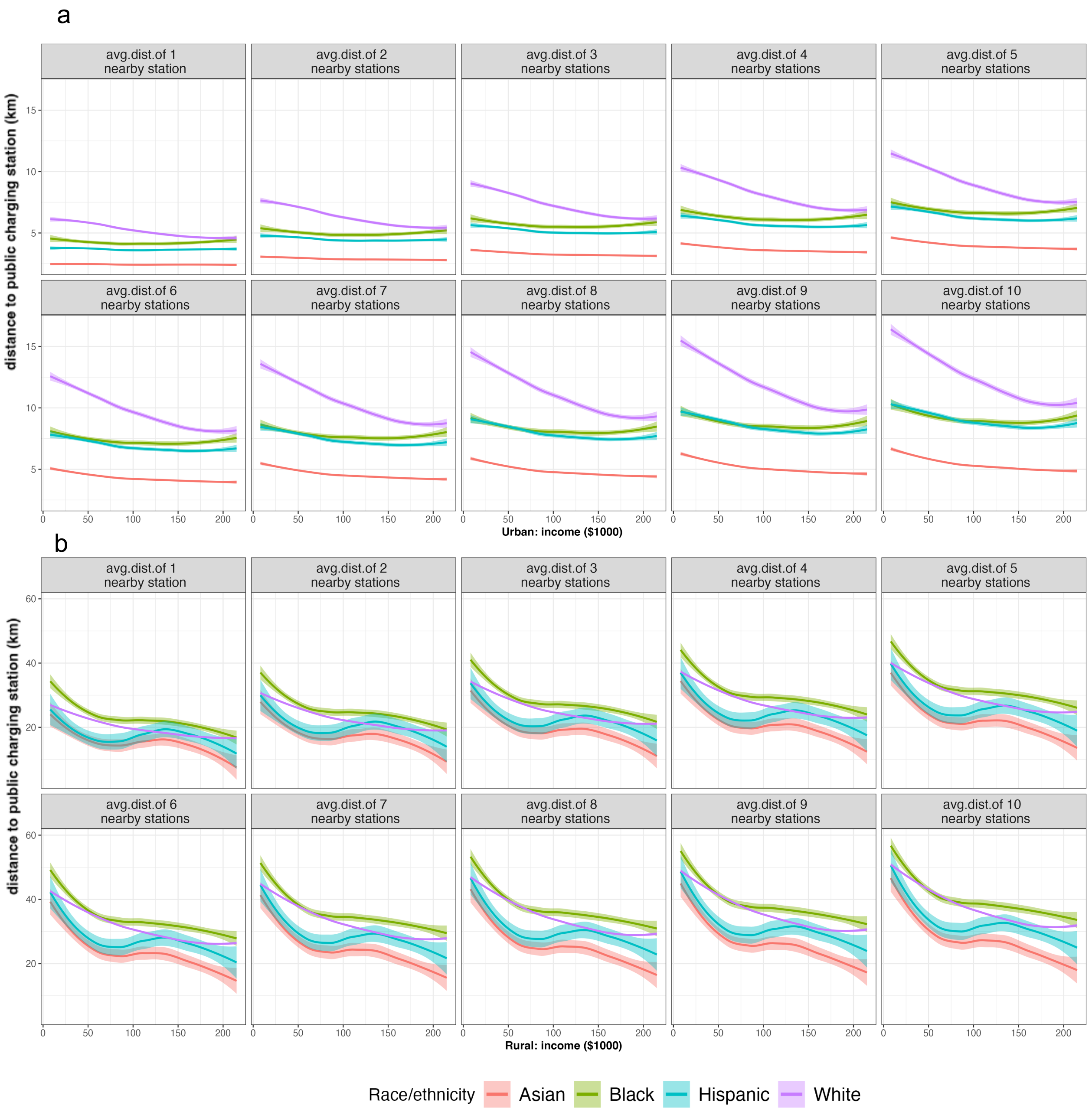
a
avg.dist.of 1 nearby station
avg.dist.of 2 nearby stations
avg.dist.of 3 nearby stations
avg.dist.of 4 nearby stations
avg.dist.of 5 nearby stations
avg.dist.of 6 nearby stations
avg.dist.of 7 nearby stations
avg.dist.of 8 nearby stations
avg.dist.of 9 nearby stations
avg.dist.of 10 nearby stations
distance to public charging station (km)
Urban: income ($1000)
b
avg.dist.of 1 nearby station
avg.dist.of 2 nearby stations
avg.dist.of 3 nearby stations
avg.dist.of 4 nearby stations
avg.dist.of 5 nearby stations
avg.dist.of 6 nearby stations
avg.dist.of 7 nearby stations
avg.dist.of 8 nearby stations
avg.dist.of 9 nearby stations
avg.dist.of 10 nearby stations
distance to public charging station (km)
Rural: income ($1000)
Race/ethnicity
Asian
Black
Hispanic
White

Supplementary Figure 2. Locally weighted scatterplot smoothing (LOESS) regression curves by average distance among k-nearest electric vehicle (EV) infrastructure in a refined sample (remove those households that had their income in the top and bottom 5%). a. LOESS in urban setting. b. LOESS in rural setting. 1 means the nearest EV infrastructure. 10 means in total 10 nearby EV stations. EV public infrastructure accessibility showing the LOESS regression curves by $100,000 interval of the entire sample (n = 107,177,734). LOESS regression curve, represented by the solid line in the center, shows the predictive value, while the shadow around it represents the 95% confidence interval.

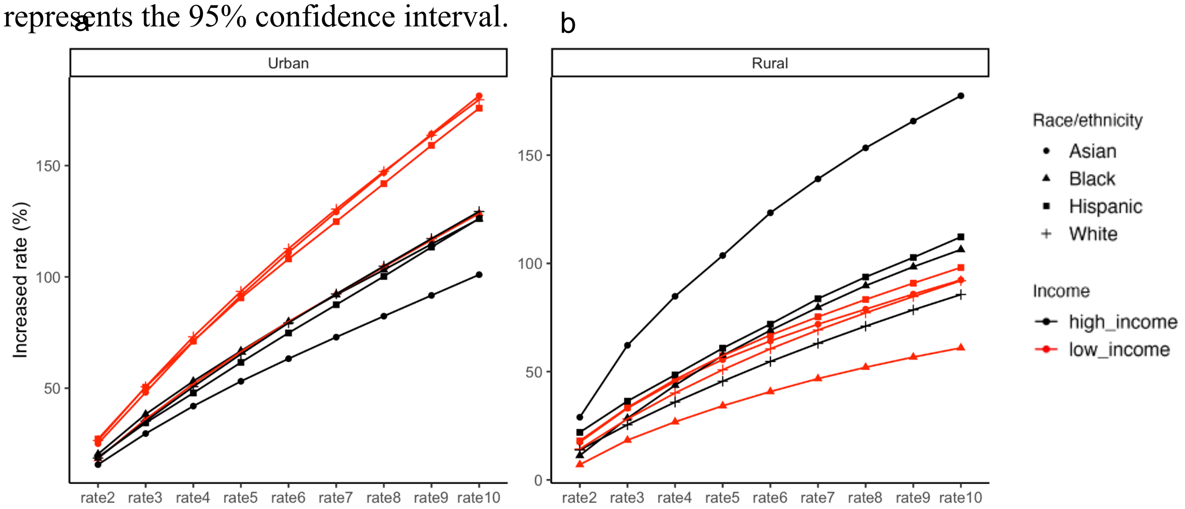

Supplementary Figure 3. Increased rates (%) between Nth and (N-1)th nearest electric vehicle (EV) infrastructure accessibility among different racial/ethnic populations by urban and rural in a refined sample (remove those households that had their income in the top and bottom 5%). a. Increased rates in urban setting. b. Increased rate in rural setting.

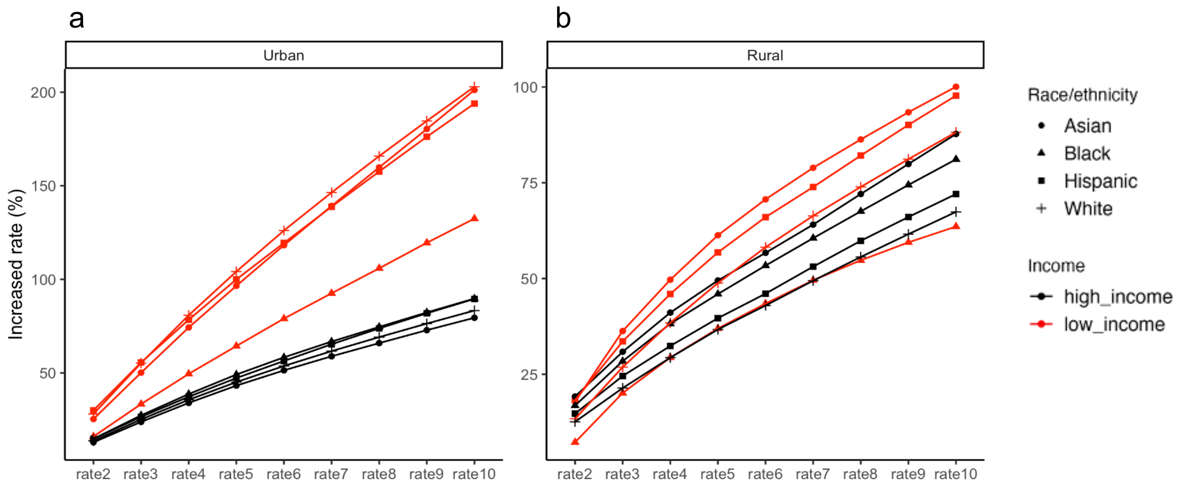

Supplementary Figure 4. Increased rates (%) between Nth and (N-1)th nearest electric vehicle (EV) infrastructure accessibility among different racial/ethnic populations by urban and rural. a. Increased rates in urban setting. b. Increased rate in rural setting.

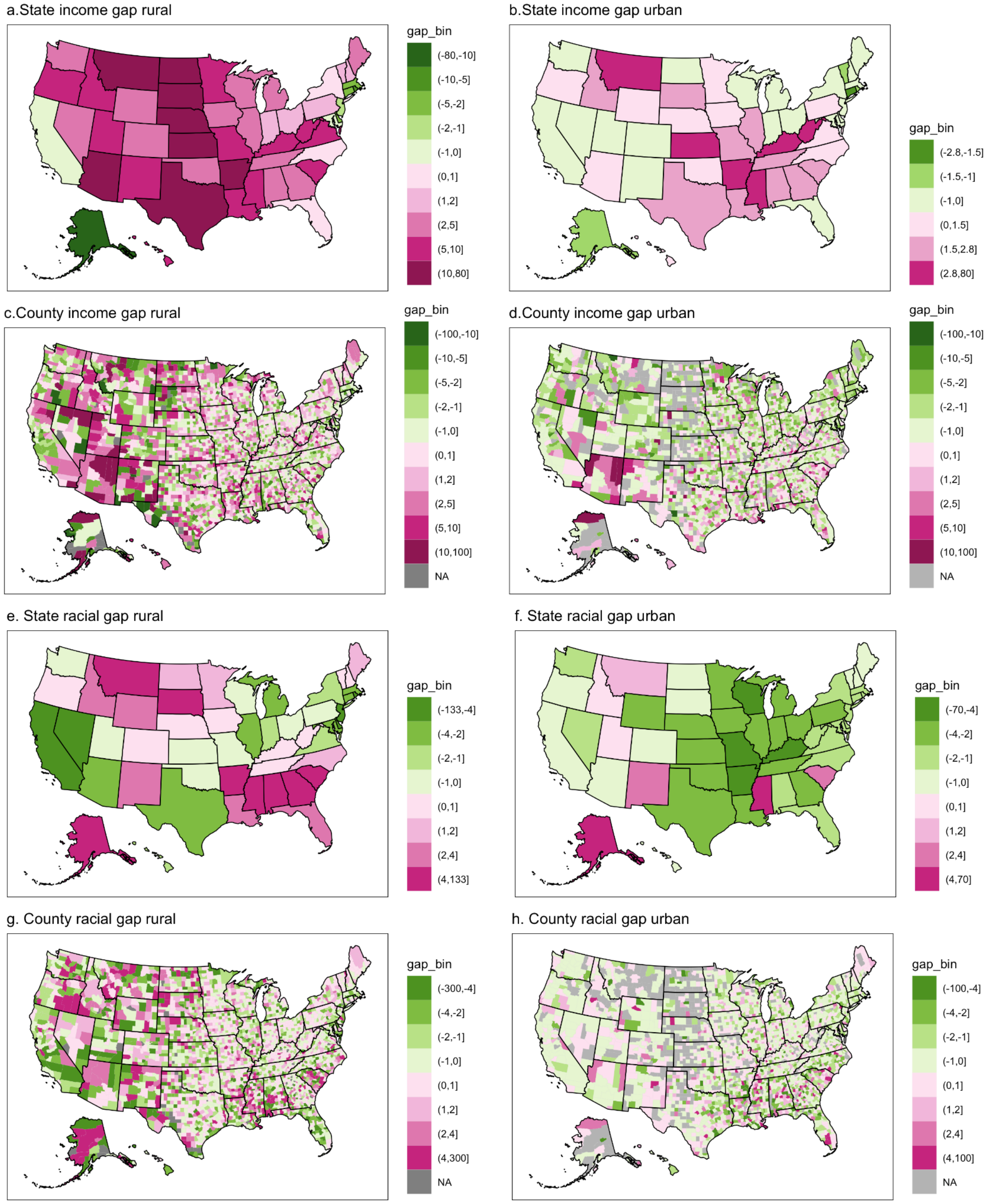

Supplementary Figure 5. Equity assessment through accessibility gaps at state and county level using DP2. DP2 means the average distance of the 2 nearest electric vehicle (EV) stations. a-d: accessibility gaps between low-moderate income **(**LMI) and non-LMI households by their location at state and county; e-h: accessibility gaps between Black and White households by their location at state and county. The magenta color scheme shows more greater gaps, and the green

color scheme shows less gaps. Gray color means no data available or beyond the defined minimum and maximum thresholds of -100 and 100, respectively.

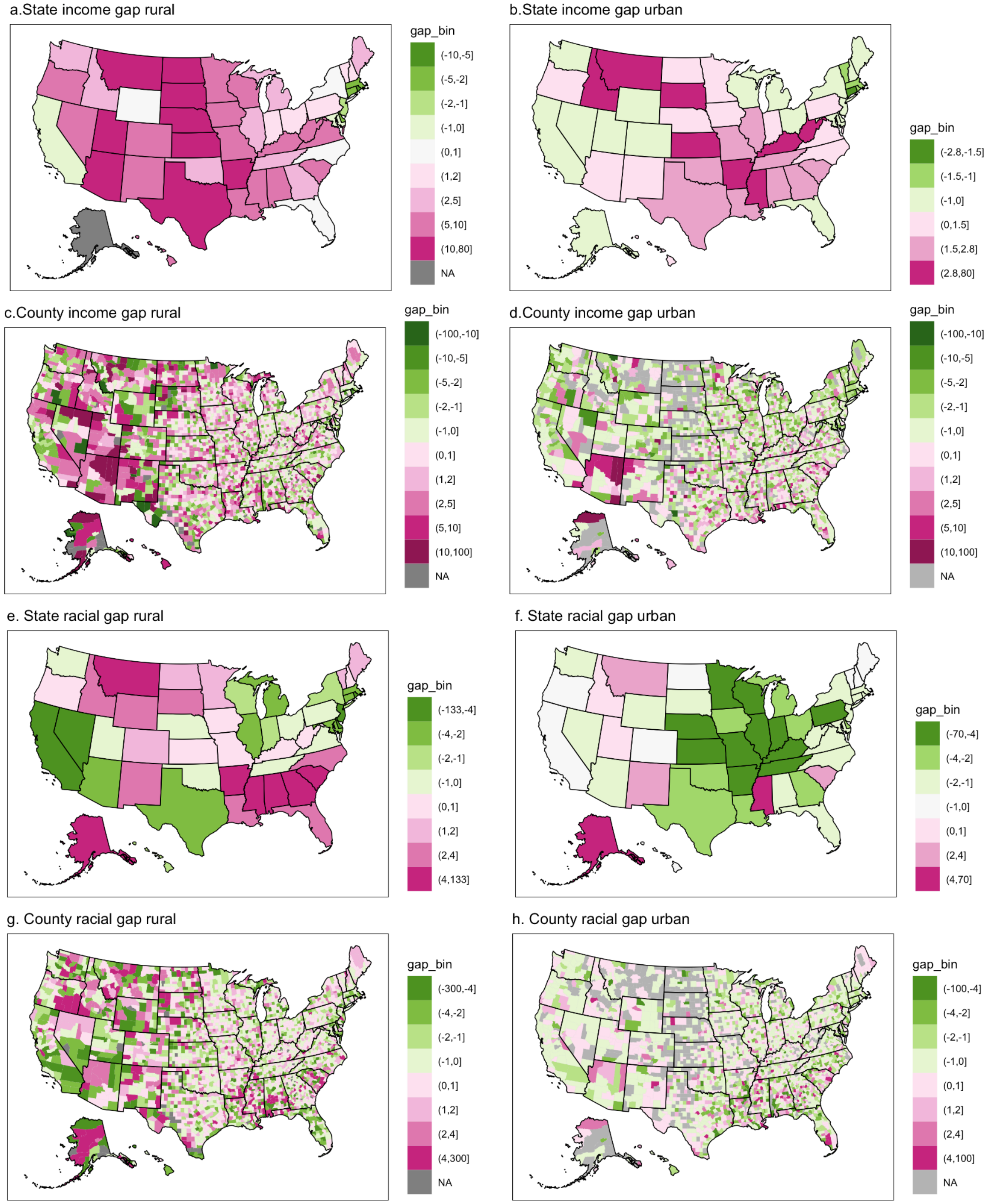

Supplementary Figure 6. Equity assessment through accessibility gaps at state and county level using DP3. DP3 means the average distance of the 3 nearest electric vehicle (EV) stations. a-d: accessibility gaps between low-moderate income (LMI) and non-LMI households by their location at state and county; e-h: accessibility gaps between Black and White households by their location at state and county. The magenta color scheme shows more greater gaps, and the green

color scheme shows less gaps. Gray color means no data available or beyond the defined minimum and maximum thresholds of -100 and 100, respectively.

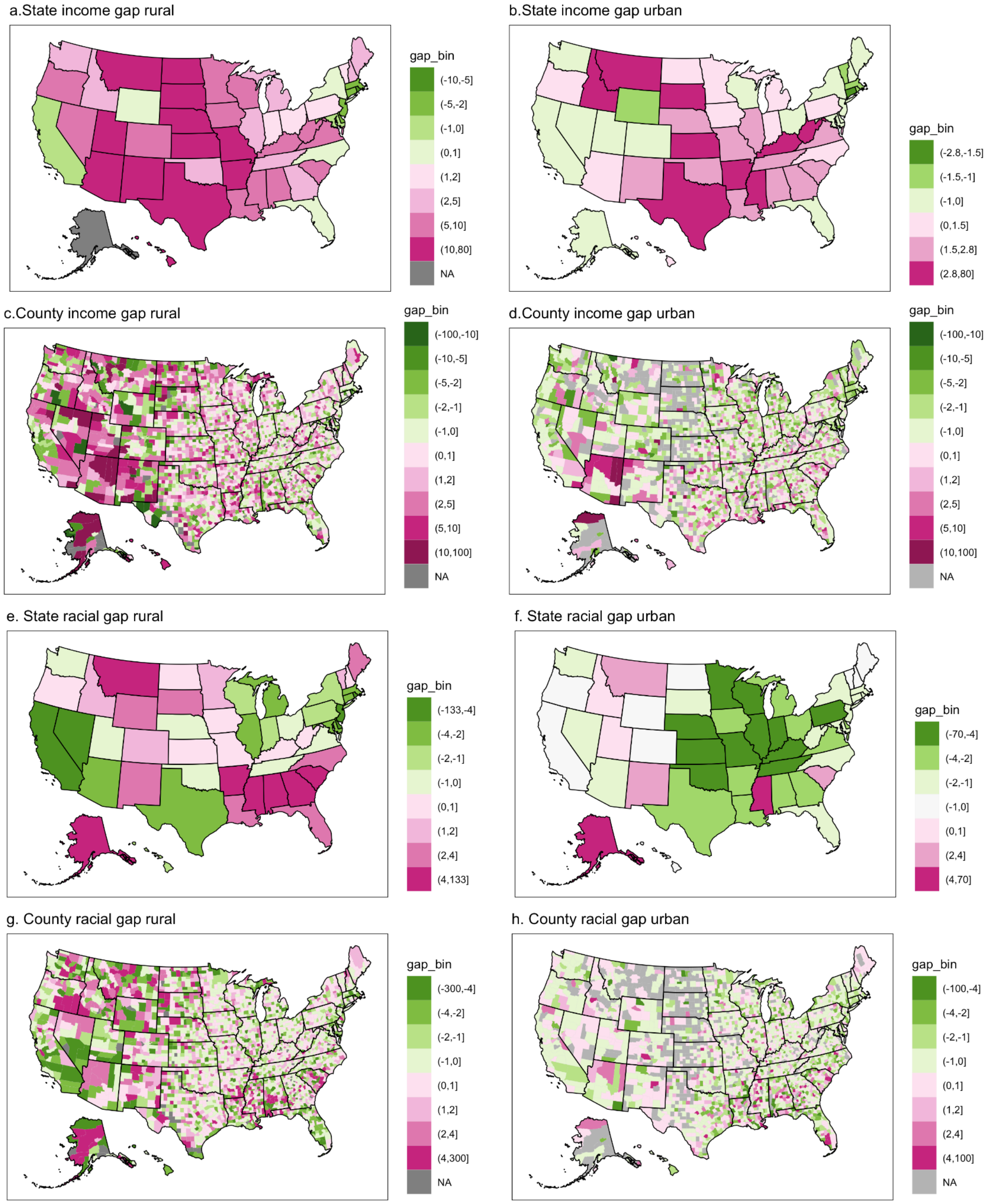

Supplementary Figure 7. Equity assessment through accessibility gaps at state and county level using DP4. DP4 means the average distance of the 4 nearest electric vehicle (EV) stations. a-d: accessibility gaps between low-moderate income (LMI) and non-LMI households by their location at state and county; e-h: accessibility gaps between Black and White households by their location at state and county. The magenta color scheme shows more greater gaps, and the green

color scheme shows less gaps. Gray color means no data available or beyond the defined minimum and maximum thresholds of -100 and 100, respectively.

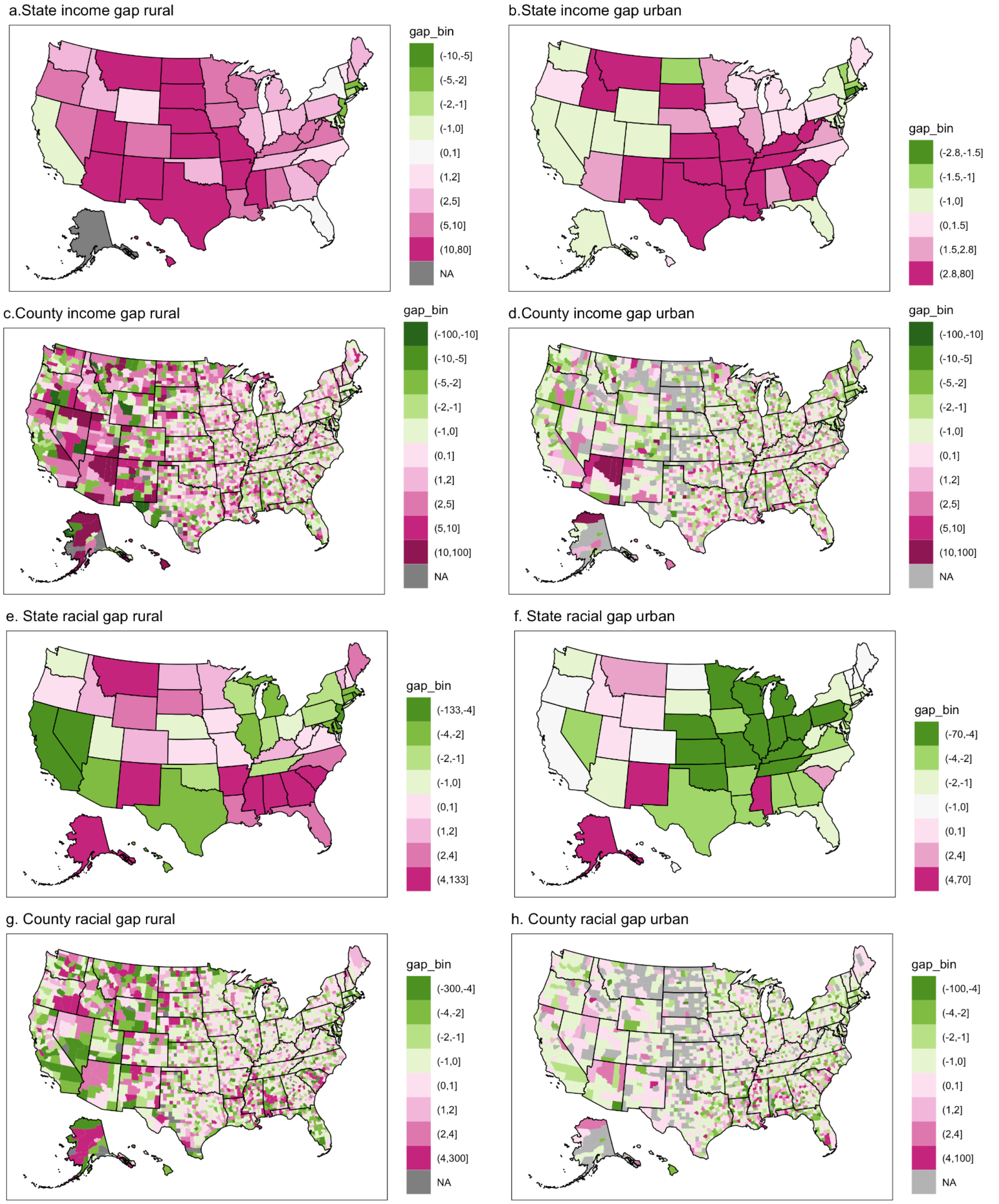

Supplementary Figure 8. Equity assessment through accessibility gaps at state and county level using DP5. DP5 means the average distance of the 5 nearest electric vehicle (EV) stations. a-d: accessibility gaps between low-moderate income **(**LMI) and non-LMI households by their location at state and county; e-h: accessibility gaps between Black and White households by their location at state and county. The magenta color scheme shows more greater gaps, and the green

color scheme shows less gaps. Gray color means no data available or beyond the defined minimum and maximum thresholds of -100 and 100, respectively.

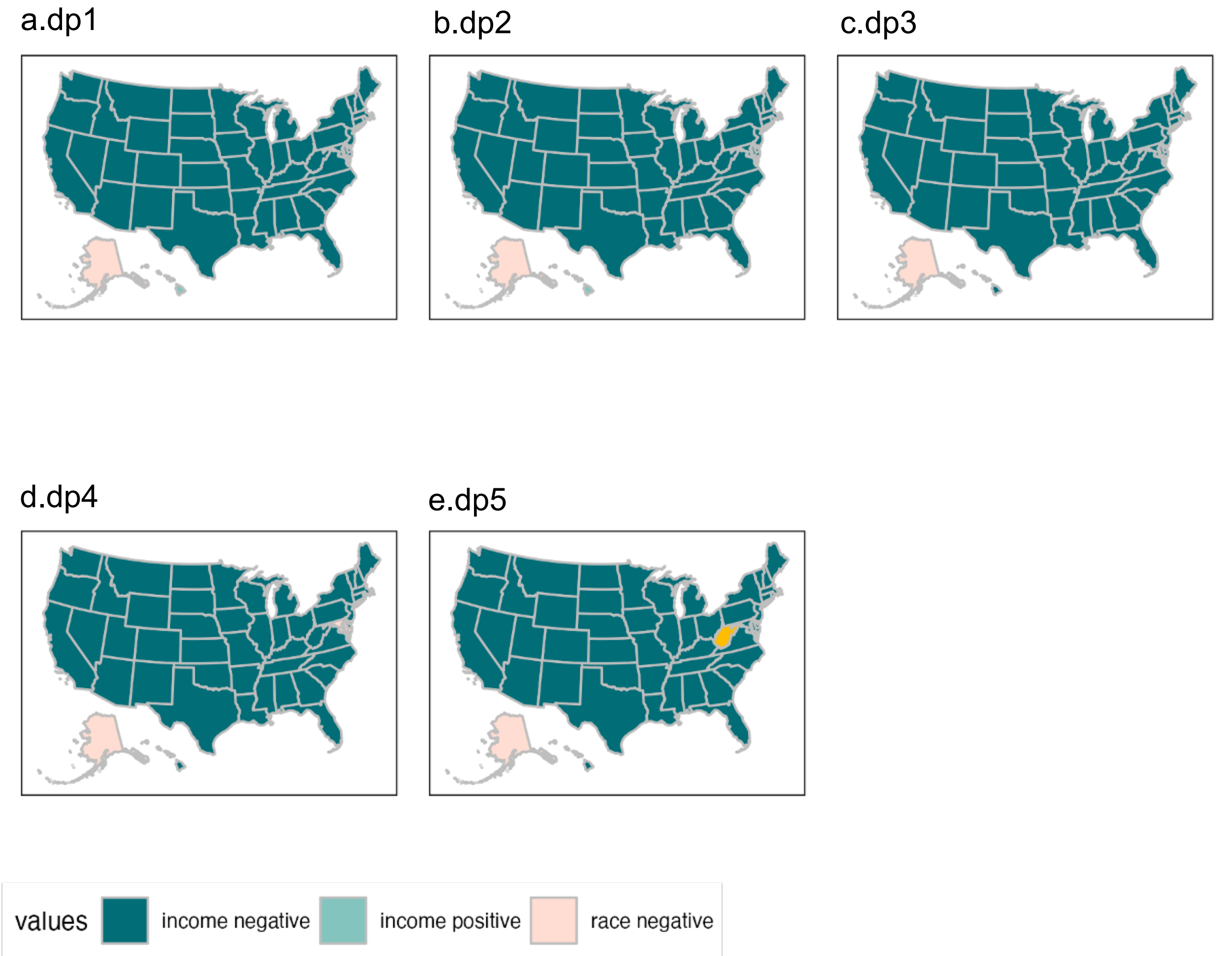

Supplementary Figure 9. State level least absolute shrinkage and selection operator (LASSO) coefficients for rural single-house dwelling residents for all five outcomes (dp1-dp5). a-e: LASSO outcome from dp1 to dp5. Dp1 means the distance to the nearest station. Dp5 means the average distance of the 5 nearest electric vehicle (EV) stations. Coefficients are color-coded to denote their direction, whether positive or negative. In the context of rural single-house dwelling residents, a negative coefficient estimate for income implies that lower-income households are linked with greater distances to the nearest EV infrastructure, whereas a positive coefficient estimate indicates the converse. Similarly, a negative coefficient estimate for race suggests that Black households are associated with shorter distances to the nearest EV infrastructure, whereas a positive coefficient estimate indicates the opposite. Consistency across all five outcomes is notably observed.

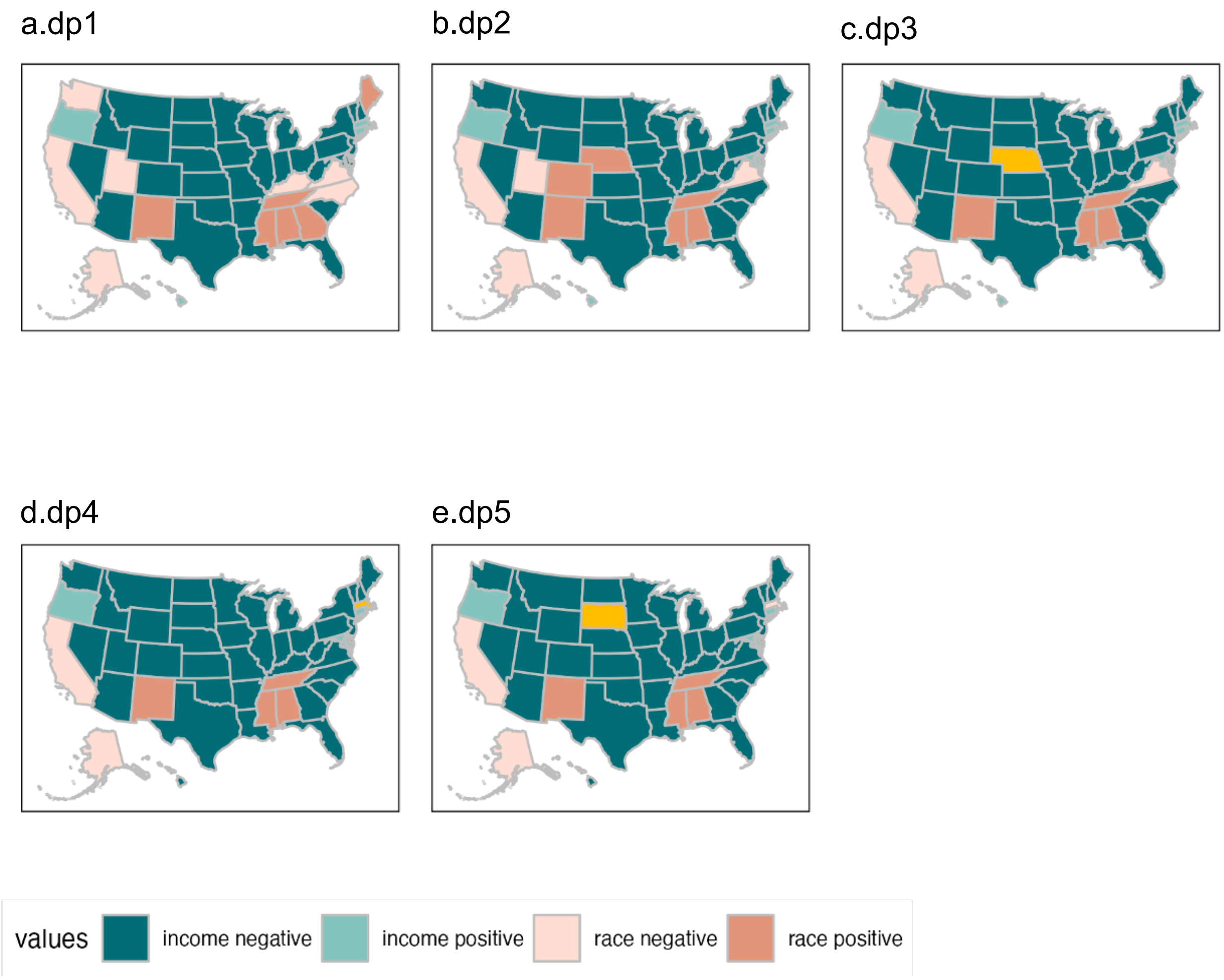

Supplementary Figure 10. State level least absolute shrinkage and selection operator (LASSO) coefficients for rural multi-unit dwelling residents for all five outcomes (dp1-dp5). a-e: LASSO outcome from dp1 to dp5. Dp1 means the distance to the nearest station. Dp5 means the average distance of the 5 nearest electric vehicle (EV) stations. Coefficients are color-coded to denote their direction, whether positive or negative. In the context of rural multi-unit dwelling residents, a negative coefficient estimate for income implies that lower-income households are linked with greater distances to the nearest EV infrastructure, whereas a positive coefficient estimate indicates the converse. Similarly, a negative coefficient estimate for race suggests that Black households are associated with shorter distances to the nearest EV infrastructure, whereas a positive coefficient estimate indicates the opposite. Consistency across all five outcomes is notably observed.

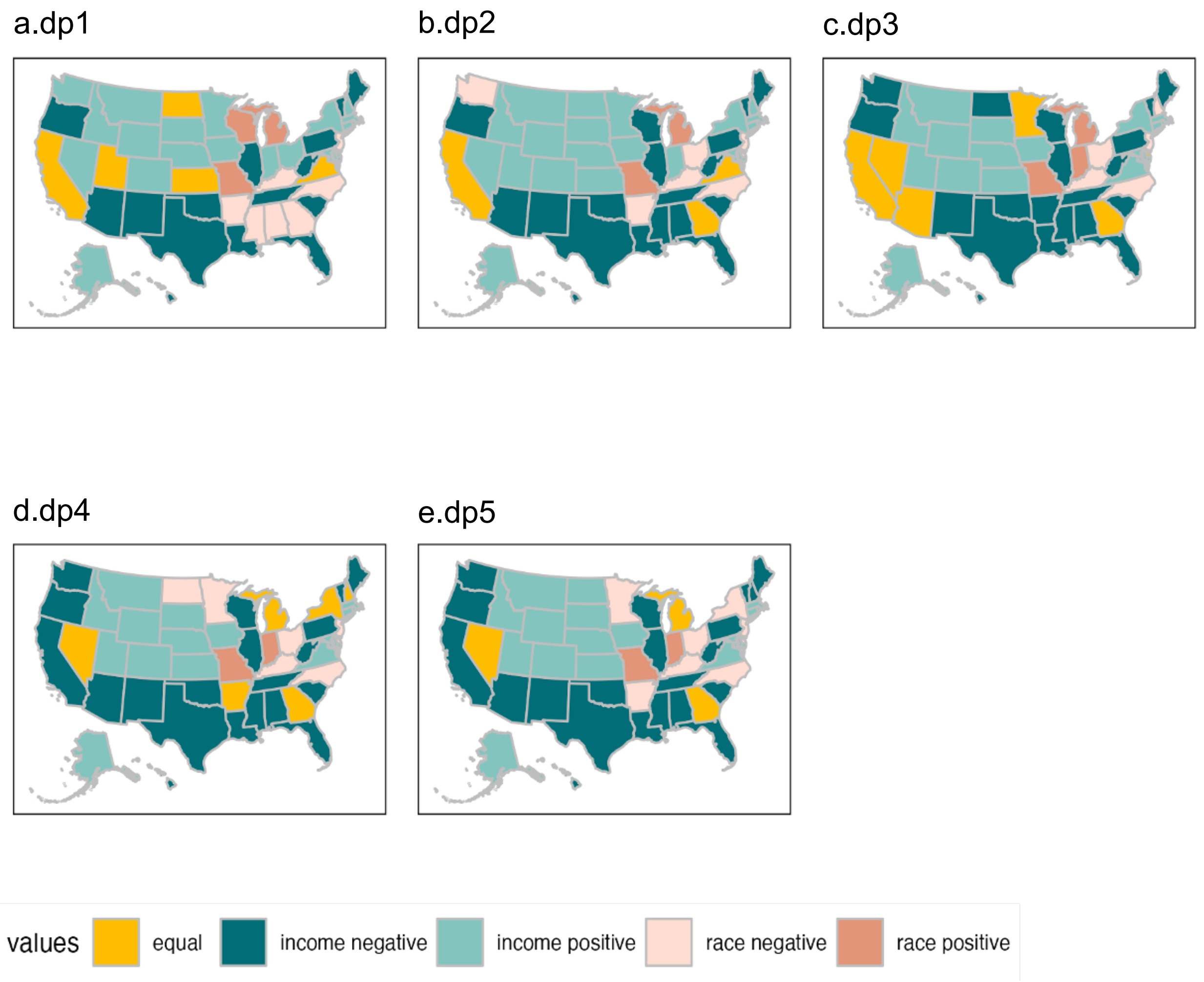

Supplementary Figure 11. State level least absolute shrinkage and selection operator (LASSO) coefficients for urban single-house dwelling residents for all five outcomes (dp1-dp5). a-e: LASSO outcome from dp1 to dp5. Dp1 means the distance to the nearest station. Dp5 means the average distance of the 5 nearest electric vehicle (EV) stations. Coefficients are color-coded to denote their direction, whether positive or negative. In the context of urban single-house dwelling residents, a negative coefficient estimate for income implies that lower-income households are linked with greater distances to the nearest EV infrastructure, whereas a positive coefficient estimate indicates the converse. Similarly, a negative coefficient estimate for race suggests that Black households are associated with shorter distances to the nearest EV infrastructure, whereas a positive coefficient estimate indicates the opposite. Consistency across all five outcomes is notably observed.

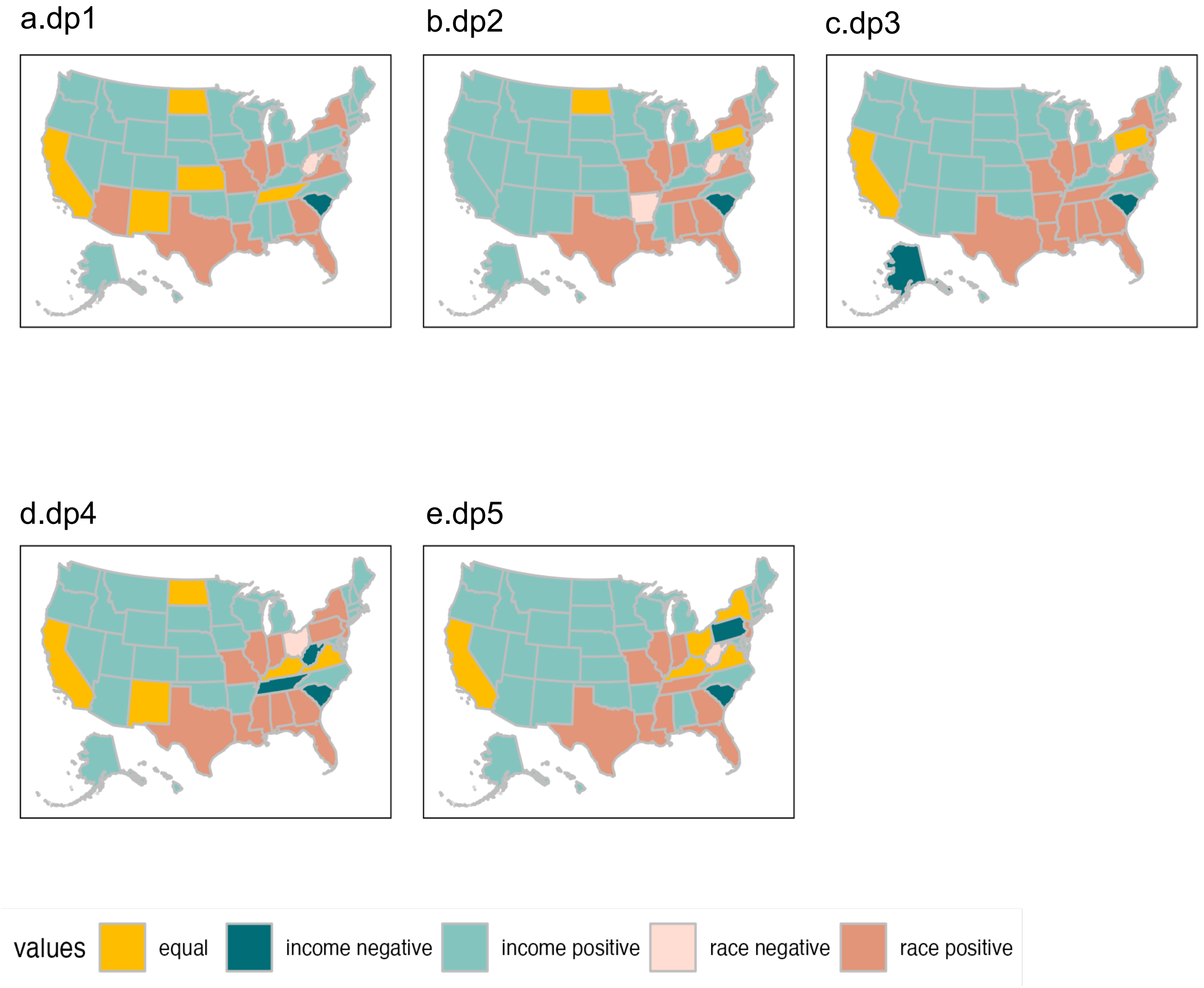

Supplementary Figure 12. State level least absolute shrinkage and selection operator (LASSO) coefficients for urban multi-unit dwelling residents for all five outcomes (dp1-dp5). a-e: LASSO outcome from dp1 to dp5. Dp1 means the distance to the nearest station. Dp5 means the average distance of the 5 nearest electric vehicle (EV) stations. Coefficients are color-coded to denote their direction, whether positive or negative. In the context of urban multi-unit dwelling residents, a negative coefficient estimate for income implies that lower-income households are linked with greater distances to the nearest EV infrastructure, whereas a positive coefficient estimate indicates the converse. Similarly, a negative coefficient estimate for race suggests that Black households are associated with shorter distances to the nearest EV infrastructure, whereas a positive coefficient estimate indicates the opposite. Consistency across all five outcomes is notably observed.

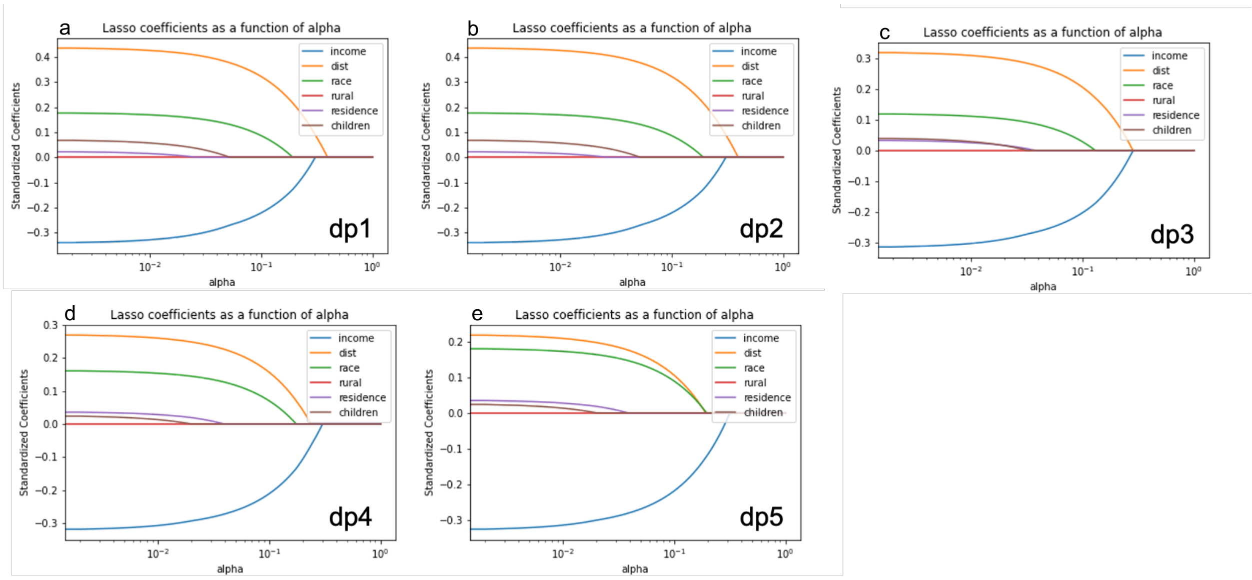

Supplementary Figure 13. Least absolute shrinkage and selection operator (LASSO) shrinkage of coefficients for Georgia (dp1-dp5). a-e: LASSO outcomes from dp1 to dp5 for Georgia. Dp1 means the distance to the nearest station. Dp5 means the average distance of the 5 nearest EV station. Each colored curve represents coefficients as a function of the alpha (the penalty, L1 norm). Note: if alpha = 0, the LASSO yields the same results as the least squares fit, and as alpha increases (from left to right in the plot), the LASSO gives the null model in which the coefficient estimates approximate towards zero. County fixed effects are residualized in all the figures.

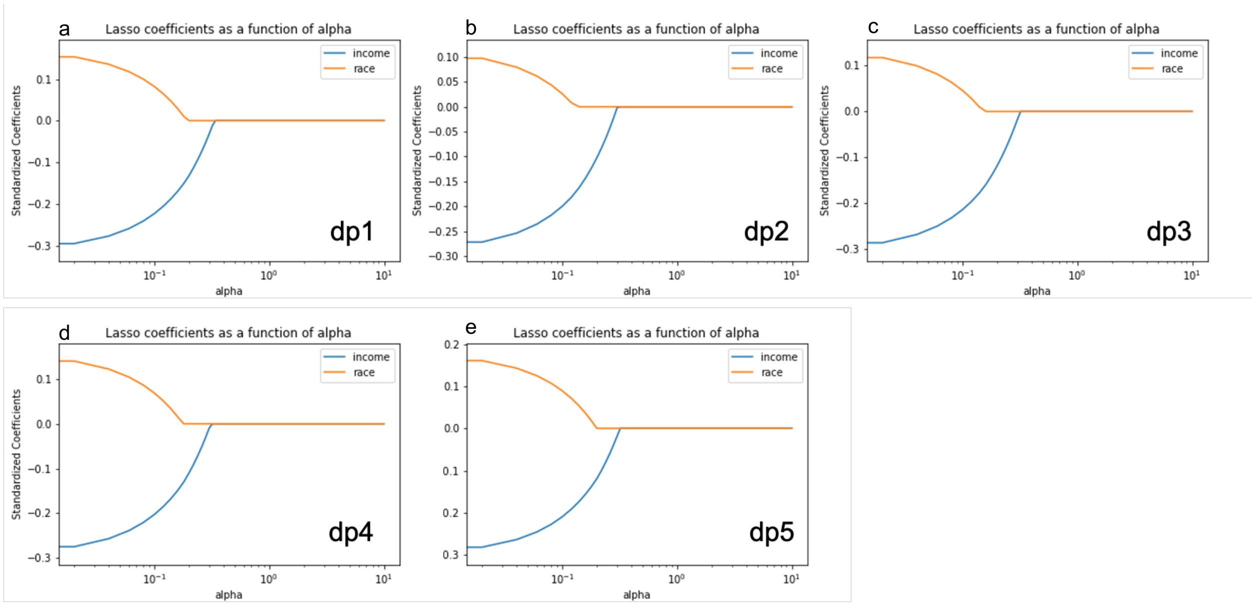

Supplementary Figure 14. Simplified least absolute shrinkage and selection operator (LASSO) shrinkage of coefficients for Georgia (dp1-dp5). a-e: Simplified LASSO outcomes from dp1 to dp5 for Georgia. Dp1 means the distance to the nearest station. Dp5 means the average distance of the 5 nearest EV stations. Each colored curve represents coefficients as a function of the alpha (the penalty, L1 norm). Note: if alpha = 0, the LASSO yields the same results as the least squares fit, and as alpha increases (from left to right in the plot), the LASSO gives the null model in which the coefficient estimates approximate towards zero. County fixed effects, distance to the highway (dist), number of children (children), duration of residence (residence) are all residualized in all the figures.

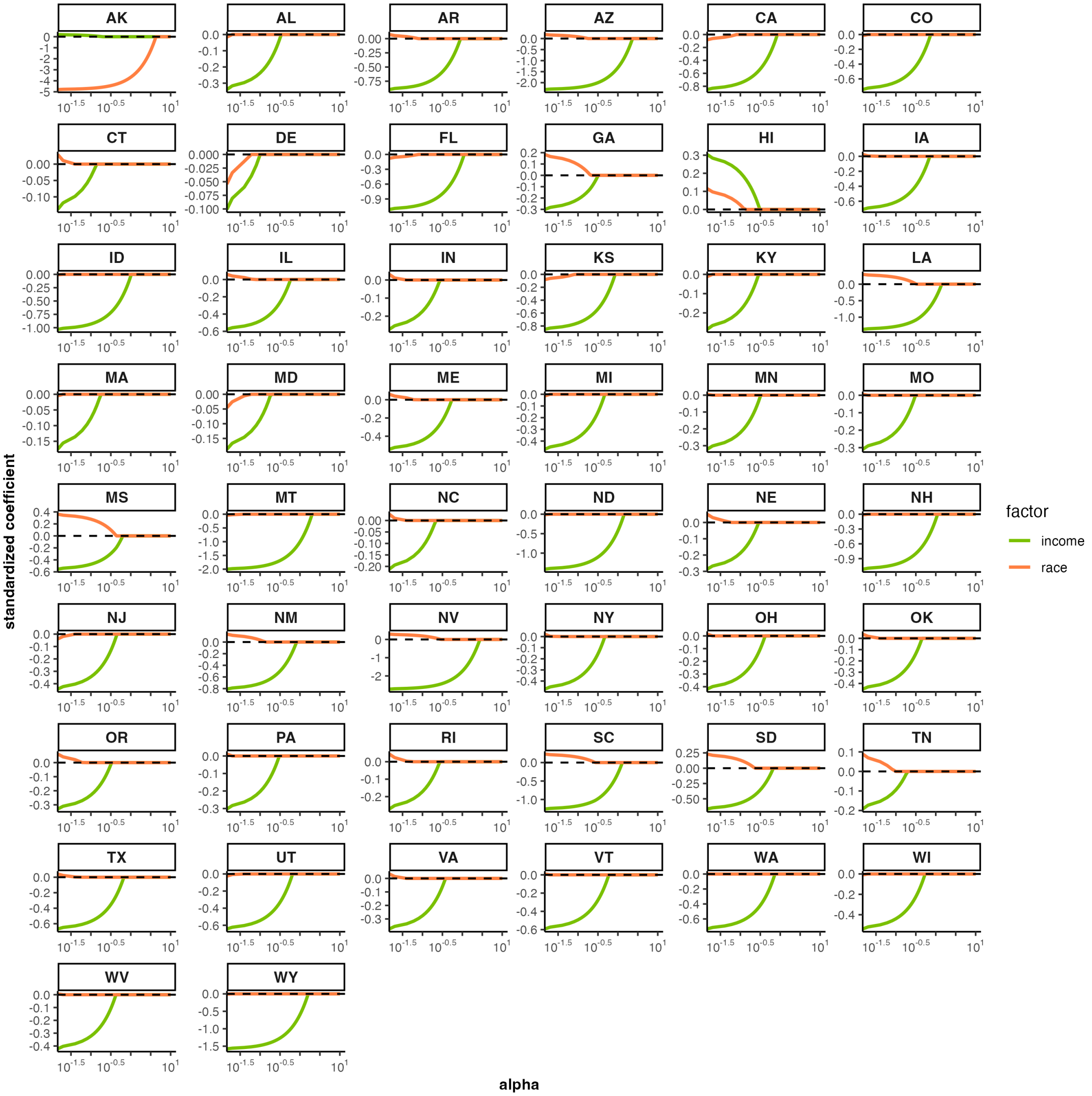

Supplementary Figure 15. State level least absolute shrinkage and selection operator (LASSO) coefficients for rural single-family dwelling (SFD) residents (dp1). Dp1 means the distance to the nearest station. Across the majority of states, income emerges as a more influential factor compared to race. Each colored curve represents coefficients as a function of the alpha (the penalty, L1 norm). Note: if alpha = 0, the LASSO yields the same results as the least squares fit, and as alpha increases (from left to right in the plot), the LASSO gives the null model in which the coefficient estimates approximate towards zero. County fixed effects, distance to the highway (dist), number of children (children), duration of residence (residence) are all residualized in all the figures. State name abbreviation note： Alabama (AL), Alaska (AK), Arizona (AZ), Arkansas (AR), California (CA), Colorado (CO), Connecticut (CT), Delaware (DE), Florida (FL), Georgia (GA), Hawaii (HI), Idaho (ID), Illinois (IL), Indiana (IN), Iowa (IA), Kansas (KS), Kentucky (KY),

Louisiana (LA), Maine (ME), Maryland (MD), Massachusetts (MA), Michigan (MI), Minnesota (MN), Mississippi (MS), Missouri (MO), Montana (MT), Nebraska (NE), Nevada (NV), New Hampshire (NH), New Jersey (NJ), New Mexico (NM), New York (NY), North Carolina (NC), North Dakota (ND), Ohio (OH), Oklahoma (OK), Oregon (OR), Pennsylvania (PA), Rhode Island (RI), South Carolina (SC), South Dakota (SD), Tennessee (TN), Texas (TX), Utah (UT), Vermont (VT), Virginia (VA), Washington (WA), West Virginia (WV), Wisconsin (WI), Wyoming (WY), and the District of Columbia (DC).

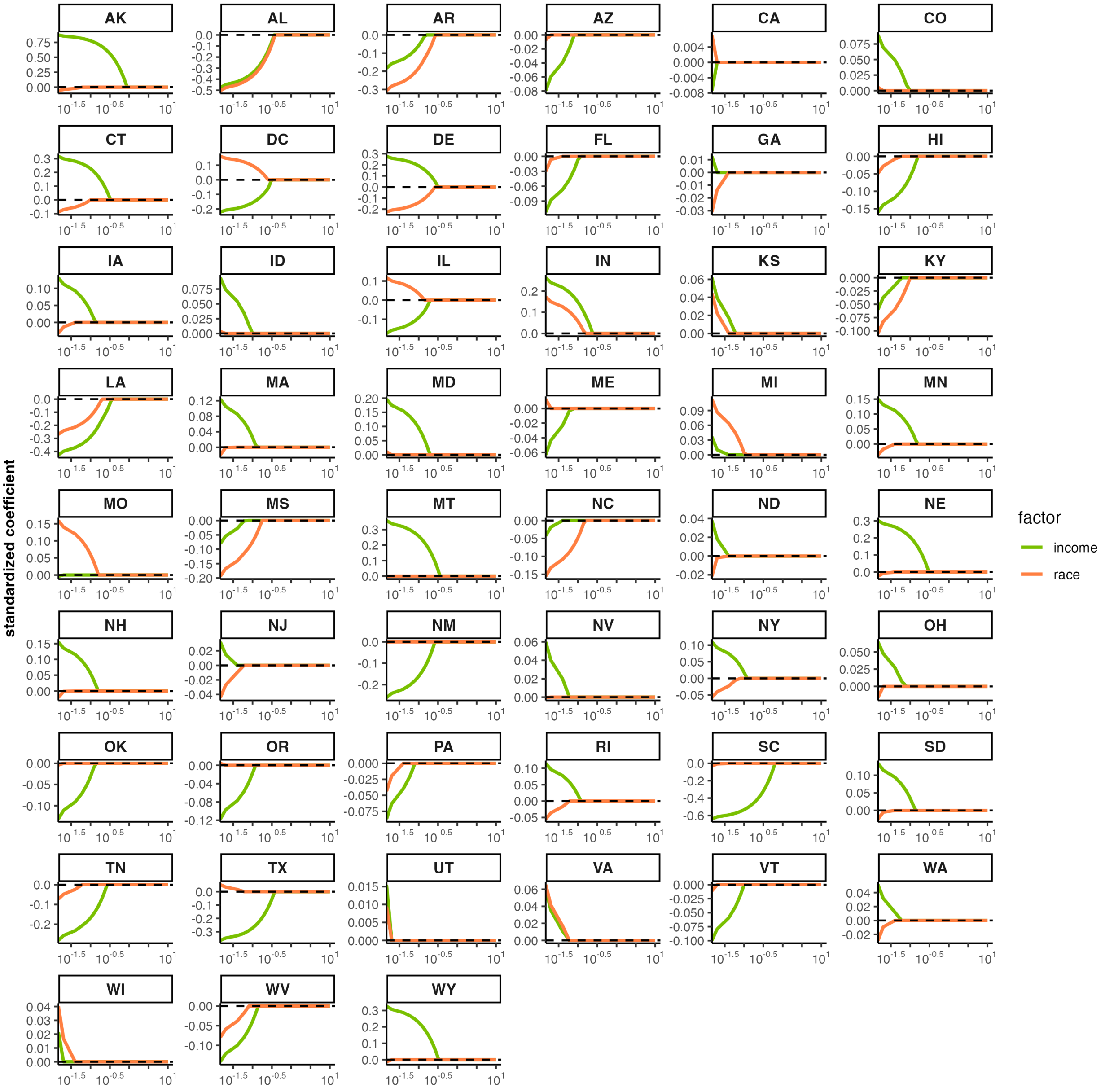

Supplementary Figure 16. State level least absolute shrinkage and selection operator (LASSO) coefficients for urban single-family dwelling (SFD) residents (dp1). Dp1 means the distance to the nearest station. Across the majority of states, income emerges as a more influential factor compared to race. Each colored curve represents coefficients as a function of the alpha (the penalty, L1 norm). Note: if alpha = 0, the LASSO yields the same results as the least squares fit, and as alpha increases (from left to right in the plot), the LASSO gives the null model in which the coefficient estimates approximate towards zero. County fixed effects, distance to the highway (dist), number of children (children), duration of residence (residence) are all residualized in all the figures. State name abbreviation note: Alabama (AL), Alaska (AK), Arizona (AZ), Arkansas (AR), California (CA), Colorado (CO), Connecticut (CT), Delaware (DE), Florida (FL), Georgia (GA), Hawaii (HI), Idaho (ID), Illinois (IL), Indiana (IN), Iowa (IA), Kansas (KS), Kentucky (KY), Louisiana (LA), Maine (ME), Maryland (MD), Massachusetts (MA), Michigan (MI), Minnesota

(MN), Mississippi (MS), Missouri (MO), Montana (MT), Nebraska (NE), Nevada (NV), New Hampshire (NH), New Jersey (NJ), New Mexico (NM), New York (NY), North Carolina (NC), North Dakota (ND), Ohio (OH), Oklahoma (OK), Oregon (OR), Pennsylvania (PA), Rhode Island (RI), South Carolina (SC), South Dakota (SD), Tennessee (TN), Texas (TX), Utah (UT), Vermont (VT), Virginia (VA), Washington (WA), West Virginia (WV), Wisconsin (WI), Wyoming (WY), and the District of Columbia (DC).

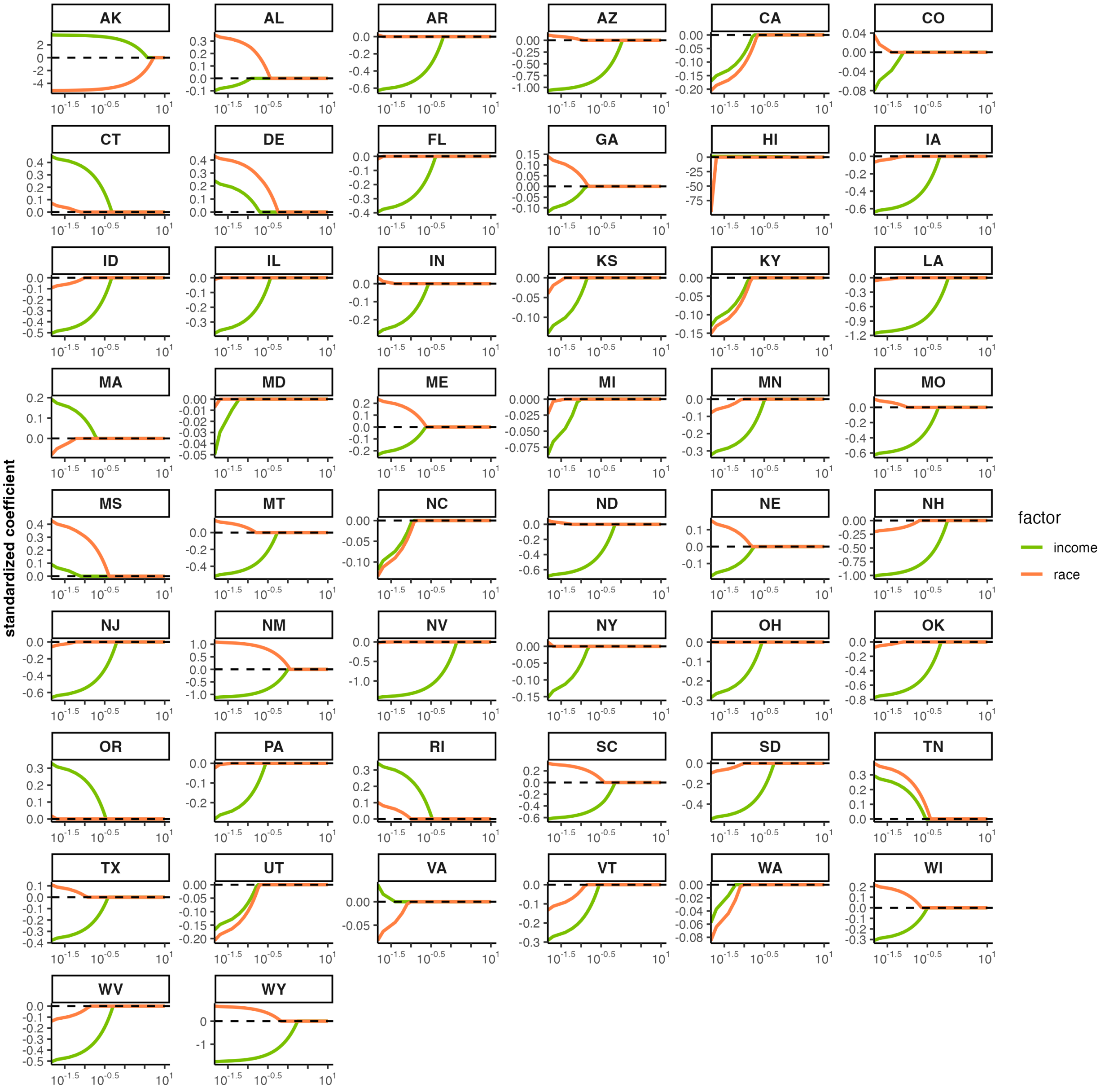

Supplementary Figure 17. State level least absolute shrinkage and selection operator (LASSO) coefficients for rural multi-unit dwelling (MUD) residents (dp1). Dp1 means the distance to the nearest station. Across many states, income emerges as a more influential factor compared to race. However, we start to observe that race plays a more dominant role compared to the income in a few states. Each colored curve represents coefficients as a function of the alpha (the penalty, L1 norm). Note: if alpha = 0, the LASSO yields the same results as the least squares fit, and as alpha increases (from left to right in the plot), the LASSO gives the null model in which the coefficient estimates approximate towards zero. County fixed effects, distance to the highway (dist), number of children (children), duration of residence (residence) are all residualized in all the figures. State name abbreviation note: Alabama (AL), Alaska (AK), Arizona (AZ), Arkansas (AR), California (CA), Colorado (CO), Connecticut (CT), Delaware (DE), Florida (FL), Georgia

(GA), Hawaii (HI), Idaho (ID), Illinois (IL), Indiana (IN), Iowa (IA), Kansas (KS), Kentucky (KY), Louisiana (LA), Maine (ME), Maryland (MD), Massachusetts (MA), Michigan (MI), Minnesota (MN), Mississippi (MS), Missouri (MO), Montana (MT), Nebraska (NE), Nevada (NV), New Hampshire (NH), New Jersey (NJ), New Mexico (NM), New York (NY), North Carolina (NC), North Dakota (ND), Ohio (OH), Oklahoma (OK), Oregon (OR), Pennsylvania (PA), Rhode Island (RI), South Carolina (SC), South Dakota (SD), Tennessee (TN), Texas (TX), Utah (UT), Vermont (VT), Virginia (VA), Washington (WA), West Virginia (WV), Wisconsin (WI), Wyoming (WY), and the District of Columbia (DC).

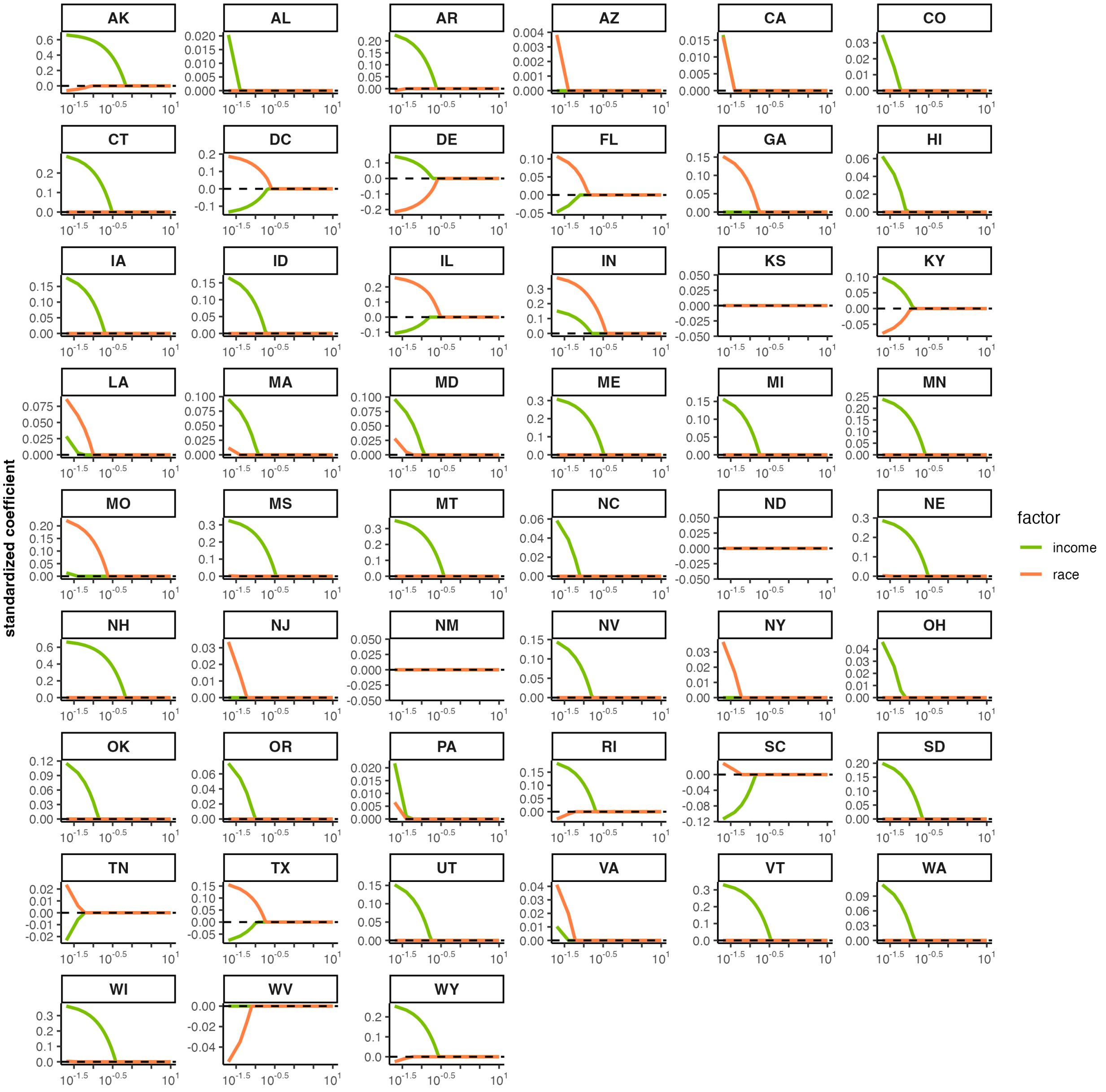

Supplementary Figure 18. State level least absolute shrinkage and selection operator (LASSO) coefficients for urban multi-unit dwelling (MUD) residents (dp1). Dp1 means the distance to the nearest station. Across many states, income emerges as a more influential factor compared to race. However, we start to observe that race plays a more dominant role compared to the income in a few states. Each colored curve represents coefficients as a function of the alpha (the penalty, L1 norm). Note: if alpha = 0, the LASSO yields the same results as the least squares fit, and as alpha increases (from left to right in the plot), the LASSO gives the null model in which the coefficient estimates approximate towards zero. County fixed effects, distance to the highway (dist), number of children (children), duration of residence (residence) are all residualized in all the figures. State name abbreviation note: Alabama (AL), Alaska (AK), Arizona (AZ), Arkansas (AR), California (CA), Colorado (CO), Connecticut (CT), Delaware (DE), Florida (FL), Georgia (GA), Hawaii (HI), Idaho (ID), Illinois (IL), Indiana (IN), Iowa (IA), Kansas (KS), Kentucky (KY),

Louisiana (LA), Maine (ME), Maryland (MD), Massachusetts (MA), Michigan (MI), Minnesota (MN), Mississippi (MS), Missouri (MO), Montana (MT), Nebraska (NE), Nevada (NV), New Hampshire (NH), New Jersey (NJ), New Mexico (NM), New York (NY), North Carolina (NC), North Dakota (ND), Ohio (OH), Oklahoma (OK), Oregon (OR), Pennsylvania (PA), Rhode Island (RI), South Carolina (SC), South Dakota (SD), Tennessee (TN), Texas (TX), Utah (UT), Vermont (VT), Virginia (VA), Washington (WA), West Virginia (WV), Wisconsin (WI), Wyoming (WY), and the District of Columbia (DC).

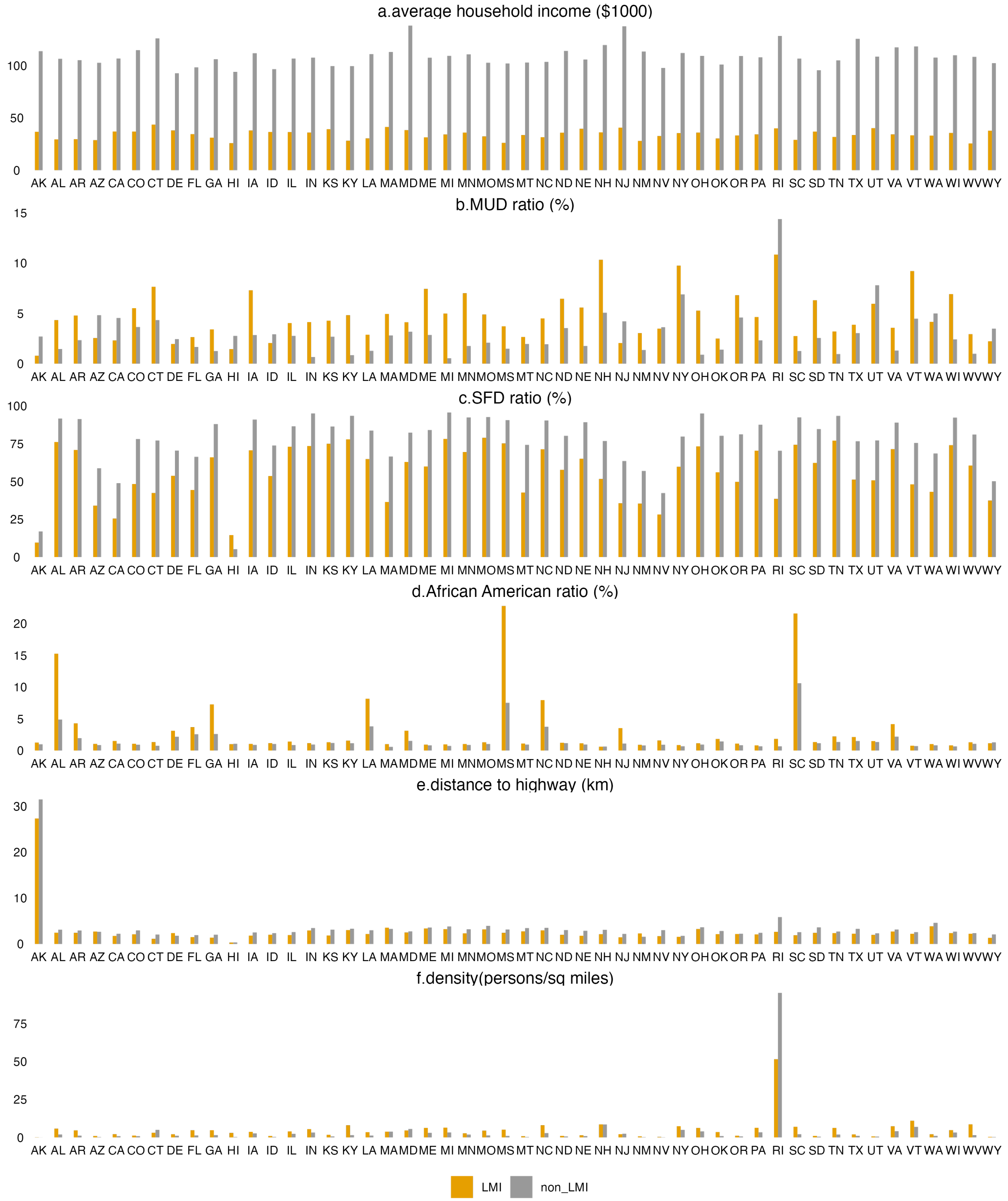

Supplementary Figure 19. Basic statistics by state in rural areas, comparing Low-to-Moderate Income (LMI) and non-LMI groups across various indicators. a. Average household income by state ($1000). b. Multi-unit dwellings (MUD) ratio by state. c. Single-family dwelling (SFD) ratio by state. d. Black population ratios by state. e. Distance to highway by state. f. Population density by state. Among these indicators, LMI households consistently exhibit higher

ratios of Black households, population densities, and MUD compared to non-LMI households across states. Conversely, LMI households tend to have lower average household incomes, distances to highways, and SFD ratios compared to non-LMI households. State abbreviation note: Alabama (AL), Alaska (AK), Arizona (AZ), Arkansas (AR), California (CA), Colorado (CO), Connecticut (CT), Delaware (DE), Florida (FL), Georgia (GA), Hawaii (HI), Idaho (ID), Illinois (IL), Indiana (IN), Iowa (IA), Kansas (KS), Kentucky (KY), Louisiana (LA), Maine (ME), Maryland (MD), Massachusetts (MA), Michigan (MI), Minnesota (MN), Mississippi (MS), Missouri (MO), Montana (MT), Nebraska (NE), Nevada (NV), New Hampshire (NH), New Jersey (NJ), New Mexico (NM), New York (NY), North Carolina (NC), North Dakota (ND), Ohio (OH), Oklahoma (OK), Oregon (OR), Pennsylvania (PA), Rhode Island (RI), South Carolina (SC), South Dakota (SD), Tennessee (TN), Texas (TX), Utah (UT), Vermont (VT), Virginia (VA), Washington (WA), West Virginia (WV), Wisconsin (WI), Wyoming (WY), and the District of Columbia (DC).

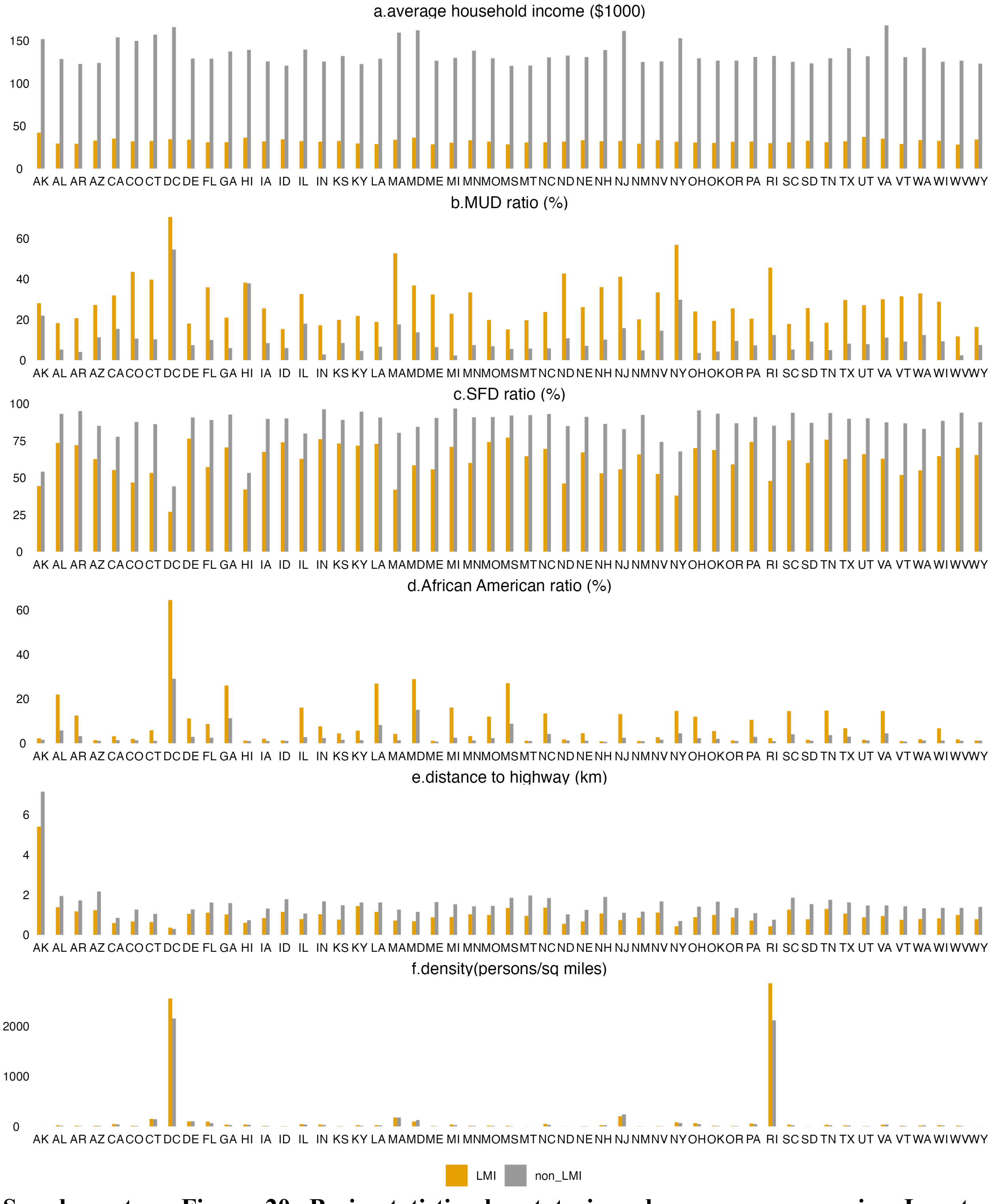

Supplementary Figure 20. Basic statistics by state in urban areas, comparing Low-to-Moderate Income (LMI) and non-LMI groups across various indicators. a. Average household income by state ($1000). b. Multi-unit dwellings (MUD) ratio by state. c. Single-family dwelling (SFD) ratio by state. d. Black population ratios by state. e. Distance to highway by state. f. Population density by state. Among these indicators, LMI households consistently exhibit higher

ratios of Black residents, population densities, and MUD compared to non-LMI households across states. Conversely, LMI households tend to have lower average household incomes, distances to highways, and SFD ratios compared to non-LMI households. State name abbreviation note: Alabama (AL), Alaska (AK), Arizona (AZ), Arkansas (AR), California (CA), Colorado (CO), Connecticut (CT), Delaware (DE), Florida (FL), Georgia (GA), Hawaii (HI), Idaho (ID), Illinois (IL), Indiana (IN), Iowa (IA), Kansas (KS), Kentucky (KY), Louisiana (LA), Maine (ME), Maryland (MD), Massachusetts (MA), Michigan (MI), Minnesota (MN), Mississippi (MS), Missouri (MO), Montana (MT), Nebraska (NE), Nevada (NV), New Hampshire (NH), New Jersey (NJ), New Mexico (NM), New York (NY), North Carolina (NC), North Dakota (ND), Ohio (OH), Oklahoma (OK), Oregon (OR), Pennsylvania (PA), Rhode Island (RI), South Carolina (SC), South Dakota (SD), Tennessee (TN), Texas (TX), Utah (UT), Vermont (VT), Virginia (VA), Washington (WA), West Virginia (WV), Wisconsin (WI), Wyoming (WY), and the District of Columbia (DC).

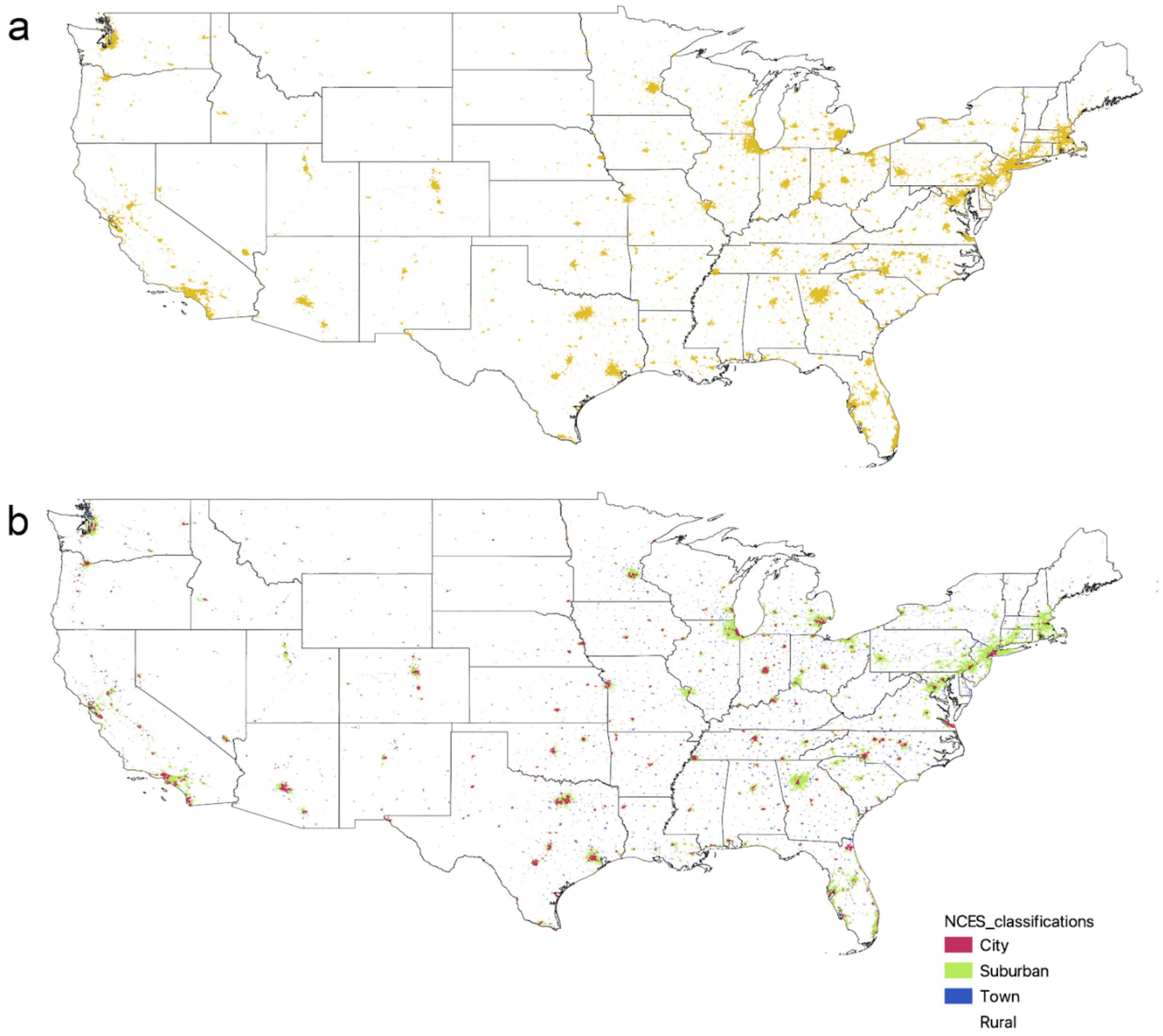

Supplementary Figure 21. Comparison of the urban and rural definitions from the Census to the National Center for Education Statistics (NCES). a.2020 Census Urban Areas. b. 2021 NCES Locale Classification. The definition of urban from Census aligned seamlessly with the town, suburban, and city categories suggested by the (NCES) definition.

Supplementary Table 1. Electric Vehicle (EV) Infrastructure Accessibility for United States Households by Each State, Panel A

STATE	Household average distance to the EV infrastructure (km)	Household average distance to the nearest 2 public charging stations	Household average distance to the nearest 3 public charging stations	Household average distance to the nearest 4 public charging stations	Household average distance to the nearest 5 public charging stations
AK	102.31	115.81	122.96	129.17	133.98
AL	15.14	16.84	18.44	20.48	22.08
AR	19.26	22.24	26.43	29.43	31.99
AZ	5.97	6.99	8.16	9.20	10.07
CA	1.98	2.29	2.56	2.81	3.04
CO	3.30	4.09	4.73	5.30	5.88
CT	2.82	3.36	3.79	4.17	4.53
DC	0.97	1.19	1.34	1.48	1.61
DE	3.73	4.15	4.66	5.10	5.59
FL	4.29	4.97	5.56	6.05	6.53
GA	6.59	7.81	8.78	9.64	10.42
HI	2.40	3.10	3.69	4.23	4.64
IA	12.11	14.73	17.08	19.18	21.32
ID	11.50	15.40	18.92	23.05	26.50
IL	7.35	8.61	9.73	10.58	11.32
IN	9.22	11.00	12.71	14.22	15.51
KS	12.95	16.91	19.48	21.77	24.31
KY	14.83	18.24	21.20	23.45	25.46
LA	13.27	15.24	17.01	18.89	20.85
MA	2.17	2.51	2.82	3.10	3.36
MD	2.42	2.86	3.27	3.68	4.05
ME	8.29	9.73	10.97	12.22	13.50
MI	6.52	7.93	9.00	10.00	11.03
MN	10.83	12.94	15.07	16.99	18.72
MO	10.74	12.19	13.50	14.77	16.00
MS	22.01	24.72	28.50	32.10	34.77
MT	31.17	39.00	46.66	52.92	57.21
NC	5.90	7.08	8.13	9.05	9.93
ND	21.91	24.40	28.92	32.57	37.62
NE	11.61	13.75	16.59	18.95	21.28

NH	7.62	9.26	10.44	11.34	12.14
NJ	3.07	3.68	4.19	4.66	5.09
NM	17.09	22.91	26.93	30.30	33.48
NV	4.17	5.26	6.07	6.67	7.32
NY	2.88	3.45	3.92	4.34	4.71
OH	5.08	6.05	6.90	7.74	8.49
OK	9.36	11.36	13.39	15.72	17.78
OR	4.42	5.49	6.46	7.53	8.41
PA	6.06	7.18	8.24	9.19	10.04
RI	2.26	2.57	2.83	3.06	3.30
SC	8.89	10.36	11.59	12.81	14.11
SD	26.25	35.42	41.97	48.98	55.10
TN	9.99	12.14	13.63	14.83	15.97
TX	7.56	9.23	10.62	11.86	13.06
UT	4.03	5.28	6.56	7.40	8.10
VA	6.48	7.61	8.47	9.29	10.02
VT	5.96	7.02	8.04	8.87	9.62
WA	4.11	5.09	5.90	6.56	7.21
WI	10.87	12.96	14.93	16.98	18.63
WV	14.78	16.68	19.00	20.86	22.73
WY	20.40	23.76	32.64	47.02	58.15

Supplementary Table 1. Electric Vehicle (EV) Infrastructure Accessibility for United States Households by Each State, Panel B

STATE	Household average distance to the nearest 6 public charging stations	Household average distance to the nearest 7 public charging stations	Household average distance to the nearest 8 public charging stations	Household average distance to the nearest 9 public charging stations	Household average distance to the nearest 10 public charging stations
AK	142.47	148.84	153.80	159.12	163.56
AL	23.46	24.78	26.02	27.10	28.12
AR	34.27	36.43	38.44	40.27	41.92
AZ	10.88	11.65	12.49	13.38	14.20
CA	3.25	3.45	3.65	3.87	4.08
CO	6.39	6.87	7.34	7.83	8.27
CT	4.88	5.22	5.54	5.86	6.15
DC	1.72	1.82	1.92	2.00	2.08
DE	6.02	6.40	6.81	7.18	7.57
FL	6.98	7.38	7.76	8.13	8.50
GA	11.19	11.90	12.53	13.13	13.68
HI	5.00	5.33	5.65	5.93	6.19
IA	23.21	25.14	26.89	28.68	30.44
ID	29.27	31.67	33.63	35.75	38.21
IL	12.06	12.78	13.51	14.21	14.91
IN	16.68	17.72	18.66	19.52	20.40
KS	26.87	29.22	31.40	33.35	35.22
KY	27.46	29.26	31.04	32.76	34.46
LA	22.43	23.85	25.67	28.20	30.58
MA	3.59	3.80	4.01	4.20	4.39
MD	4.37	4.67	4.96	5.23	5.48
ME	14.63	15.71	16.71	17.66	18.62
MI	11.93	12.72	13.48	14.25	14.98
MN	20.46	22.11	23.58	24.91	26.18
MO	17.21	18.32	19.39	20.42	21.39
MS	37.93	40.60	43.22	45.53	47.94
MT	62.94	67.52	73.13	77.91	82.31
NC	10.71	11.45	12.17	12.86	13.51
ND	41.67	46.42	50.18	53.53	57.10
NE	23.37	25.24	27.22	29.03	30.69

NH	12.84	13.51	14.20	14.93	15.67
NJ	5.47	5.82	6.14	6.45	6.76
NM	36.04	38.54	40.82	43.01	45.64
NV	8.05	8.77	9.45	10.08	10.69
NY	5.04	5.35	5.64	5.92	6.18
OH	9.17	9.83	10.44	11.01	11.53
OK	19.72	21.42	23.03	24.54	25.91
OR	9.19	9.92	10.65	11.35	12.10
PA	10.82	11.55	12.20	12.80	13.36
RI	3.53	3.74	3.95	4.16	4.38
SC	15.15	16.04	16.87	17.62	18.30
SD	59.88	63.88	67.32	70.32	72.97
TN	17.13	18.20	19.20	20.14	21.14
TX	14.11	15.10	16.06	17.03	18.04
UT	8.76	9.29	9.81	10.29	10.79
VA	10.73	11.39	12.00	12.63	13.32
VT	10.42	11.13	11.78	12.39	12.97
WA	7.85	8.46	9.06	9.61	10.13
WI	20.21	21.63	22.93	24.20	25.39
WV	24.66	27.03	29.08	31.15	33.24
WY	68.59	77.44	85.18	92.32	98.47

State abbreviation note: Alabama (AL), Alaska (AK), Arizona (AZ), Arkansas (AR), California (CA), Colorado (CO), Connecticut (CT), Delaware (DE), Florida (FL), Georgia (GA), Hawaii (HI), Idaho (ID), Illinois (IL), Indiana (IN), Iowa (IA), Kansas (KS), Kentucky (KY), Louisiana (LA), Maine (ME), Maryland (MD), Massachusetts (MA), Michigan (MI), Minnesota (MN), Mississippi (MS), Missouri (MO), Montana (MT), Nebraska (NE), Nevada (NV), New Hampshire (NH), New Jersey (NJ), New Mexico (NM), New York (NY), North Carolina (NC), North Dakota (ND), Ohio (OH), Oklahoma (OK), Oregon (OR), Pennsylvania (PA), Rhode Island (RI), South Carolina (SC), South Dakota (SD), Tennessee (TN), Texas (TX), Utah (UT), Vermont (VT), Virginia (VA), Washington (WA), West Virginia (WV), Wisconsin (WI), Wyoming (WY), and the District of Columbia (DC).

Supplementary Table 2. Households Coverage of Data Axle Compared to the United States Census

State Code	State	Households in our sample (All)	Households in ACS 2021	Percentage	Percentage Bars
AL	Alabama	1913536	2286466	83.69%	
AK	Alaska	192804	322179	59.84%	
AZ	Arizona	2258473	3057351	73.87%	
AR	Arkansas	1130203	1389027	81.37%	
CA	California	12024270	14336516	83.87%	
CO	Colorado	2141841	2448201	87.49%	
CT	Connecticut	1368356	1531765	89.33%	
DE	Delaware	376913	442608	85.16%	
DC	District of	281806	320408	87.95%	
FL	Florida	8276174	9622262	86.01%	
GA	Georgia	3643825	4357847	83.62%	
HI	Hawaii	368355	550892	66.87%	
ID	Idaho	573575	745488	76.94%	
IL	Illinois	4532260	5391427	84.06%	
IN	Indiana	2508988	2922060	85.86%	
IA	Iowa	1170834	1419392	82.49%	
KS	Kansas	972077	1288694	75.43%	
KY	Kentucky	1633738	2004885	81.49%	
LA	Louisiana	1825180	2091267	87.28%	
ME	Maine	536524	752215	71.33%	
MD	Maryland	2157226	2466013	87.48%	
MA	Massachusett	2664220	2935882	90.75%	
MI	Michigan	3909821	4625731	84.52%	
MN	Minnesota	1984051	2470516	80.31%	
MS	Mississippi	1007269	1341502	75.09%	
MO	Missouri	2229686	2822794	78.99%	
MT	Montana	343308	522216	65.74%	
NE	Nebraska	668353	851873	78.46%	
NV	Nevada	1096505	1280869	85.61%	
NH	New	519246	643311	80.71%	
NJ	New Jersey	3177988	3634937	87.43%	
NM	New Mexico	620842	954990	65.01%	
NY	New York	7082371	8407168	84.24%	
NC	North	4121095	4734376	87.05%	
ND	North Dakota	258000	391144	65.96%	
OH	Ohio	4591712	5236344	87.69%	
OK	Oklahoma	1387797	1754643	79.09%	
OR	Oregon	1392172	1807516	77.02%	
PA	Pennsylvania	4686553	5758863	81.38%	
RI	Rhode Island	423287	471845	89.71%	
SC	South	1891590	2344207	80.69%	
SD	South Dakota	287738	402810	71.43%	
TN	Tennessee	2457711	3015080	81.51%	
TX	Texas	9806733	11249880	87.17%	
UT	Utah	926237	1124641	82.36%	
VT	Vermont	249386	340568	73.23%	
VA	Virginia	3008216	3559067	84.52%	
WA	Washington	2457760	3191666	77.01%	
WV	West Virginia	617112	897130	68.79%	
WI	Wisconsin	2456426	2722118	90.24%	
WY	Wyoming	180395	282879	63.77%	

State abbreviation note: Alabama (AL), Alaska (AK), Arizona (AZ), Arkansas (AR), California (CA), Colorado (CO), Connecticut (CT), Delaware (DE), Florida (FL), Georgia (GA), Hawaii (HI), Idaho (ID), Illinois (IL), Indiana (IN), Iowa (IA), Kansas (KS), Kentucky (KY), Louisiana (LA), Maine (ME), Maryland (MD), Massachusetts (MA), Michigan (MI), Minnesota (MN), Mississippi (MS), Missouri (MO), Montana (MT), Nebraska (NE), Nevada (NV), New Hampshire (NH), New Jersey (NJ), New Mexico (NM), New York (NY), North Carolina (NC), North Dakota (ND), Ohio (OH), Oklahoma (OK), Oregon (OR), Pennsylvania (PA), Rhode Island (RI), South Carolina (SC), South Dakota (SD), Tennessee (TN), Texas (TX), Utah (UT), Vermont (VT), Virginia (VA), Washington (WA), West Virginia (WV), Wisconsin (WI), Wyoming (WY), and the District of Columbia (DC).

Supplementary Table 3. Locally Weighted Scatterplot Smoothing (LEOSS) Income Bin Breakdown

Panel A. Rural

	Income bin Unit: $1000	Race/Ethnicity	Rural	Distance to nearest charging station	Income range	Distance to nearest 10 charging stations	Diff.	Growth rate (%)
**1**	(4.505,5.99]	Asian	Rural	28.06964	low_income (5)	56.154845	28.0852	100.05543
**2**	(4.505,5.99]	Black	Rural	41.70655	low_income (5)	68.217363	26.51082	63.56512
**3**	(4.505,5.99]	Hispanic	Rural	25.09416	low_income (5)	49.620824	24.52666	97.7385
**4**	(4.505,5.99]	White	Rural	29.04457	low_income (5)	54.657966	25.6134	88.18653
**5**	(499,500.5]	Asian	Rural	4.003916	High_income (500)	7.516182	3.512266	87.72078
**6**	(499,500.5]	Black	Rural	5.752916	High_income (500)	10.419072	4.666156	81.10941
**7**	(499,500.5]	Hispanic	Rural	5.671056	High_income (500)	9.757054	4.085998	72.05004
**8**	(499,500.5]	White	Rural	6.615386	High_income (500)	11.074061	4.458675	67.39855

Panel B. Urban

	Income bin Unit: $1000	Race/Ethnicity	Urban	Distance to nearest charging station	Income range	Distance to nearest 10 charging stations	Diff.	Growth rate (%)
**1**	(4.505,5.99]	Asian	Urban	1.814905	low_income (5)	5.466227	3.651322	201.18529
**2**	(4.505,5.99]	Black	Urban	4.897698	low_income (5)	11.385477	6.487779	132.46589
**3**	(4.505,5.99]	Hispanic	Urban	3.216482	low_income (5)	9.454063	6.237581	193.92555
**4**	(4.505,5.99]	White	Urban	5.001857	low_income (5)	15.15232	10.15046	202.93387
**5**	(499,500.5]	Asian	Urban	2.393222	High_income (500)	4.295315	1.902093	79.47831
**6**	(499,500.5]	Black	Urban	2.827291	High_income (500)	5.364834	2.537542	89.7517
**7**	(499,500.5]	Hispanic	Urban	2.621929	High_income (500)	4.970025	2.348096	89.55604
**8**	(499,500.5]	White	Urban	2.833594	High_income (500)	5.195238	2.361644	83.34449

Supplementary Table 4. Correlation Between Data Axle and United States Census Datasets on Each Population

State	White Correlation	Black Correlation	Asian Correlation	Hispanic Correlation	Median Income Correlation
**AK**	0.95921055	0.85417152	0.94341309	0.933331447	0.650338893
**AL**	0.97226049	0.83857593	0.92548254	0.90788594	0.739773581
**AR**	0.97935182	0.83325368	0.79906114	0.986814867	0.503363626
**AZ**	0.98244511	0.89289398	0.90215464	0.977313712	0.866999318
**CA**	0.91107358	0.81698053	0.96376628	0.938220878	0.850046622
**CT**	0.97312768	0.82870866	0.91826388	0.982643448	0.905292049
**CO**	0.98817383	0.79480691	0.95532187	0.985198088	0.788291031
**DC**	0.83035865	0.92837955	0.95216129	0.96497744	0.937075619
**DE**	0.96411262	0.74476667	0.96257101	0.97142568	0.846878665
**FL**	0.93878324	0.83692158	0.89622577	0.980598313	0.891604302
**GA**	0.95066254	0.85235879	0.96965762	0.964424956	0.806079114
**HI**	0.94449117	0.7333961	0.97171689	0.84862715	0.773746233
**IA**	0.996035	0.8785852	0.93679651	0.96901931	0.63493101
**ID**	0.99423471	0.59778137	0.90061455	0.960839836	0.516256413
**IL**	0.94311378	0.95632385	0.96453579	0.975202279	0.813318408
**IN**	0.94263393	0.8091931	0.92359559	0.92468539	0.694513277
**KS**	0.99188782	0.77148381	0.92616198	0.986693721	0.767812856
**KY**	0.99183502	0.79289235	0.93440749	0.94819756	0.730818574
**LA**	0.90419869	0.87167619	0.8857016	0.864130638	0.622425158
**MA**	0.97791094	0.86015681	0.97633224	0.958523917	0.875005924
**ME**	0.99165067	0.67790671	0.81294636	0.784868957	0.634087267
**MD**	0.96798561	0.92526082	0.98040517	0.980578405	0.832056883
**MI**	0.98300834	0.94542536	0.94460346	0.968921821	0.842791593
**MN**	0.99370619	0.69912427	0.97239665	0.961681252	0.84640361
**MO**	0.98863276	0.89989212	0.94413208	0.940409632	0.758367483
**MS**	0.97241157	0.82451601	0.88345721	0.898870231	0.610623333
**MT**	0.99162515	0.74296009	0.76635661	0.952193597	0.308836093
**NC**	0.96399955	0.77111384	0.96547541	0.962381484	0.771445904
**ND**	0.99653985	0.86462807	0.90264125	0.936406816	0.371413088
**NE**	0.99441589	0.80062923	0.89296506	0.984540098	0.719011097
**NH**	0.99129439	0.79255351	0.94005989	0.954410956	0.783946118
**NJ**	0.97162663	0.90736914	0.98132722	0.987234409	0.842865105

**NM**	0.99047845	0.87381818	0.90739335	0.990325253	0.501309027
**NV**	0.96465227	0.84614703	0.97462043	0.97963474	0.733591632
**NY**	0.94431246	0.95418365	0.98082119	0.985251878	0.820877417
**OH**	0.98702063	0.8603889	0.94363887	0.965347255	0.782136038
**OK**	0.98780637	0.77026938	0.93650129	0.971335038	0.665797164
**OR**	0.98366543	0.76627578	0.95442581	0.978467986	0.663268808
**PA**	0.98710603	0.93678978	0.95529588	0.991716011	0.736423789
**RI**	0.97982131	0.95205724	0.90087865	0.982443047	0.72645938
**SC**	0.9723242	0.76022032	0.87929295	0.908331476	0.817105445
**SD**	0.99332905	0.648128	0.83283405	0.917605672	0.612321045
**TN**	0.98473539	0.87048789	0.93401259	0.96328216	0.845920198
**TX**	0.89863463	0.76893743	0.91570787	0.933683113	0.780055306
**UT**	0.97682944	0.58748616	0.91159731	0.977287805	0.686810014
**VA**	0.97072974	0.80078858	0.97745693	0.978646147	0.854067951
**VT**	0.97757034	0.6770943	0.89150354	0.820684032	0.582534237
**WA**	0.99072465	0.86822688	0.96906824	0.978816815	0.799902576
**WI**	0.94695403	0.91758439	0.92920591	0.964830693	0.839383333
**WV**	0.98389494	0.83456732	0.90009808	0.841460744	0.464961184
**WY**	0.99515953	0.84325299	0.74508887	0.953905359	0.449097655

State abbreviation Note: Alabama (AL), Alaska (AK), Arizona (AZ), Arkansas (AR), California (CA), Colorado (CO), Connecticut (CT), Delaware (DE), Florida (FL), Georgia (GA), Hawaii (HI), Idaho (ID), Illinois (IL), Indiana (IN), Iowa (IA), Kansas (KS), Kentucky (KY), Louisiana (LA), Maine (ME), Maryland (MD), Massachusetts (MA), Michigan (MI), Minnesota (MN), Mississippi (MS), Missouri (MO), Montana (MT), Nebraska (NE), Nevada (NV), New Hampshire (NH), New Jersey (NJ), New Mexico (NM), New York (NY), North Carolina (NC), North Dakota (ND), Ohio (OH), Oklahoma (OK), Oregon (OR), Pennsylvania (PA), Rhode Island (RI), South Carolina (SC), South Dakota (SD), Tennessee (TN), Texas (TX), Utah (UT), Vermont (VT), Virginia (VA), Washington (WA), West Virginia (WV), Wisconsin (WI), Wyoming (WY), and the District of Columbia (DC).

Supplementary Table 5. Statistical Summaries

Variable	Observation	Mean	Std. dev.	Min	Max
by income in rural					
EV accessibility gap	3,084	0.5106654	6.145197	-66.43601	175.9443
MUD gap	2,528	1.910126	4.333446	-98.1685	18.81997
SFD gap	2,967	-18.50888	12.98468	-83.02808	57.96402
Population density gap	3,084	2.663668	5.292677	-88.20988	131.6386
Income gap	3,084	-63214.34	17414.81	-181842.4	-21000
Black gap	2,744	0.9233598	3.947092	-49.02629	54.40829
Distance to highway gap	3,084	-0.0052044	0.0243432	-0.3946528	0.9900758
by income in urban					
EV accessibility gap	2,711	-0.3935331	2.4929	-28.62073	27.48642
MUD gap	2,408	10.91709	9.085397	-59.67426	53.45806
SFD gap	2,709	-17.77858	10.76509	-61.41825	58.33333
Population density gap	2,711	15.36447	171.7035	-1115.995	6038.446
Income gap	2,711	-80372.77	23665.2	-223209	-6000
Black gap	2,459	2.65815	6.21709	-20.83333	62.20093
Distance to highway gap	2,711	-0.0047036	0.0077164	-0.235874	0.0649177
by race in rural					
EV accessibility gap	3,078	-0.0185084	7.727401	-285.3454	129.6571
MUD gap	1,999	1.985748	4.159738	-29.54786	28.76016
SFD gap	2,896	-1.640206	10.89643	-51.27333	69.88304
Population density gap	3,077	-5.932101	8.260667	-192.9946	6.195947
Income gap	3,078	-4679.969	8926.55	-82103.31	57871.79
Poverty gap	2,951	5.011001	10.51463	-36.24385	83.39131
Distance to highway gap	3,078	-0.0014946	0.026428	-0.985121	0.6138474
by race in urban					
EV accessibility gap	2,618	-0.267609	2.105711	-18.08049	24.77434
MUD gap	2,367	5.130776	6.712941	-14.85757	99.62687
SFD gap	2,614	-3.151704	7.396737	-37.94024	87.5
Population density gap	2,617	-68.48554	371.6804	-15045.19	768.5798
Income gap	2,618	-13147.09	17253.11	-114605	337914.2
Poverty gap	2,504	12.13009	12.93631	-47.22222	62.90456
Distance to highway gap	2,618	-0.0014904	0.0065839	-0.0903768	0.0726881

Note: EV: electric vehicle; MUD: multi-unit dwelling; SFD: single-family dwelling; The gap is calculated by determining the difference in a specific variable between low-moderate income (LMI) and non-LMI households for "income," and between Black and White households for "race."

Supplementary Table 6. Estimates of Race/Ethnicity and Income on the Accessibility.

Accessibility (km)	Black dummy		Asian dummy		Hispanic dummy		Income ($1000)		# of observation
All sample	-1.781	***	-4.419	***	-3.073	***			113,739,381
	-2.321	***	-4.145	***	-3.406	***	-0.013	***	113,739,381
Rural	7.561	***	-5.132	***	-1.629	***			8,768,616
	6.011	***	-4.357	***	-2.184	***	-0.066	***	8,768,616
Urban	-1.042	***	-2.955	***	-1.742	***			104,970,765
	-1.303	***	-2.840	***	-1.908	***	-0.006	***	104,970,765
Rural_MUD	9.380	***	-8.665	***	-3.579	***			489,081
	8.238	***	-7.651	***	-3.460	***	-0.061	***	489,081
Rural_SFD	7.461	***	-4.651	***	-1.357	***			8,279,535
	5.861	***	-3.836	***	-1.950	***	-0.068	***	8,279,535
Urban_MUD	-0.203	***	-1.448	***	-0.839	***			24,366,206
	-0.273	***	-1.403	***	-0.881	***	-0.003	***	24,366,206
Urban_SFD	-0.886	***	-3.209	***	-1.684	***			80,604,559
	-1.393	***	-2.930	***	-1.990	***	-0.011	***	80,604,559

Note: Every row presents a separate regression. *** Significant at the 1 percent level. ** Significant at the 5 percent level. * Significant at the 10 percent level. We use the two-sided t statistical tests to determine the statistical significance of each coefficient in the model. MUD is multi-unit dwelling, and SFD is single-family dwelling. We performed regression analysis to investigate the correlation between accessibility and race/ethnicity, and also conditioning on income for each subgroup, respectively.

Supplementary Table 7. Pooled Cross Section Estimates.

Outcome: EV accessibility gap by income in rural				Outcome: EV accessibility gap by race in rural			
gap	Coefficient		P value	gap	Coefficient		P value
MUD gap	0.260	**	0.012	MUD gap	0.117		0.128
SFD gap	0.983	***	0.000	SFD gap	0.858	***	0.000
Population density gap	0.228	**	0.034	Population density gap	0.018		0.842
Income gap	-0.523	***	0.000	Income gap	-0.208	*	0.076
Black gap	0.087		0.388	Poverty gap	0.223	*	0.064
Distance to highway gap	1.205	***	0.000	Distance to highway gap	1.289	***	0.000
Number of obs	2362			Number of obs	1937		
R-squared	0.165			R-squared	0.339		

Outcome: EV accessibility gap by income in urban				Outcome: EV accessibility gap by race in urban			
gap	Coefficient		P value	gap	Coefficient		P value
MUD gap	-0.370	***	0.000	MUD gap	-0.396	***	0.000
SFD gap	0.036		0.686	SFD gap	-0.251	***	0.000
Population density gap	0.023		0.585	Population density gap	-0.019		0.604
Income gap	-0.111	*	0.068	Income gap	-0.138		0.111
Black gap	0.054		0.305	Poverty gap	-0.305	***	0.000
Distance to highway gap	0.433	***	0.000	Distance to highway gap	0.447	***	0.000
Number of obs	2348			Number of obs	2358		
R-squared	0.123			R-squared	0.083		

Note： *** Significant at the 1 percent level. ** Significant at the 5 percent level. * Significant at the 10 percent level. This table contains four OLS regressions, and we have regressed each outcome on the variables listed in the table. We use the two-sided t statistical tests to determine the statistical significance of each coefficient in the model. EV means electric vehicles; MUD is multi-unit dwelling and SFD is single-family dwelling. The gap is calculated by determining the difference in a specific variable between low-moderate income (LMI) and non-LMI households for "income," and between Black and White households for "race."

Supplementary Note 1

This supplementary note presents the robustness analysis for our core assessment of accessibility to EV charging stations. Our baseline analysis is grounded in the calculation of the shortest distance between households and the closest five public charging stations. However, we are fully aware that this seemingly straightforward measure may incorporate bias stemming from users' varying preferences for DC fast charging stations versus Level 2 charging stations versus. Consequently, we have implemented three additional distinct analyses to assess the robustness of our measurements.

Approach 1: In our examination of the nearest five public charging stations, we find that a substantial number of DC fast charging stations are included.

In our examination of the nearest five public charging stations, it is significant to emphasize that a substantial number of DC fast charging stations are included in the nearest five stations analysis. We find that households with at least one DC fast charging station in the five station subset ranges from around 30% upwards to 85% depending on the state (see Supplementary Table 8). Given the substantial representation of DC fast charging stations in our core assessment of accessibility, even with state-to-state variability, any potential bias in our findings will be small.

Supplementary Table 8. Count of Households with Direct-current (DC) Fast Charging Stations Among the Five Nearest Public Charging Stations.

State	Zero Fast	One fast station	Two fast stations	Three fast stations	Four fast stations	Five fast stations	Total number of fast charging stations	Fast charging ratio
AK	168844	79559	48110	28587	2357		158613	48%
AL	1073298	311674	287037	237041	67944	98108	1001804	48%
AR	845256	228057	80042	75123	12370	4170	399762	32%
AZ	1759040	370972	241489	109415	21885	6940	750701	30%
CA	9453366	2757110	1579831	921729	354337	242392	5855399	38%
CO	1413284	471319	251003	117034	56407	24204	919967	39%
CT	938128	305273	116271	66275	31137	119	519075	36%
DE	188853	110055	59084	26834	10020	66	206059	52%
DC	233554	50263	5133				55396	19%
FL	4941155	1715362	1215525	524567	254193	172287	3881934	44%
GA	2264335	670545	553365	319167	75045	51766	1669888	42%
HI	285752	63495	57452	24148	10454	8977	164526	37%
IA	497330	224949	224185	153001	107545	62463	772143	61%
ID	326110	113298	96440	62612	59226	11	331587	50%
IL	3282172	877274	660341	307468	84456	44835	1974374	38%
IN	1787894	591452	279698	152976	104738	80183	1209047	40%
KS	766921	126051	112348	25973	12851	9764	286987	27%
KY	1243437	276831	153637	63073	10410	9997	513948	29%
LA	1259950	662204	243343	112393	27358	24165	1069463	46%
MA	2284941	331264	121155	37660	16687	2657	509423	18%
ME	467056	97134	50273	27230	2595	2057	179289	28%
MD	1281192	475679	254842	162868	57574	17439	968402	43%
MI	2455623	669141	518063	310436	127653	47726	1673019	41%

MN	1189970	341660	297900	128860	114279	45091	927790	44%
MO	1173795	264768	394293	343080	140650	68413	1211204	51%
MS	526310	287185	283604	15482			586271	53%
MT	169595	163614	42398	27662	25597	16404	275675	62%
NC	2666368	964851	492054	200596	62556	23098	1743155	40%
ND	108429	68983	51190	36807	25475	13887	196342	64%
NE	397436	182987	92811	31195	17728	9880	334601	46%
NH	395396	100395	62492	16479	11494	10041	200901	34%
NJ	1761029	556205	481641	258072	119028	92868	1507814	46%
NM	538111	60591	59442	57636	19843	10139	207651	28%
NV	662470	277868	185647	91979	36604	32547	624645	49%
NY	6186854	1010291	548767	207064	76793	26188	1869103	23%
OH	3602964	680177	371044	142988	30080	6841	1231130	25%
OK	230767	150534	231701	286134	316191	399887	1384447	86%
OR	870384	318065	183796	115192	58921	49274	725248	45%
PA	3129526	948956	491919	258488	93898	52554	1845815	37%
RI	339207	57281	33949	11596	5252	1218	109296	24%
SC	1232054	429490	244280	98358	17051	1241	790420	39%
SD	111037	79691	58324	32123	31143	26627	227908	67%
TN	1606275	498303	244931	192892	54526	10268	1000920	38%
TX	7245189	2561381	1802681	624394	284344	64645	5337445	42%
UT	679294	181969	51879	32389	27596	20497	314330	32%
VA	1667665	656626	511588	242847	111970	26979	1550010	48%
VT	217149	60802	36272	11110	1492	242	109918	34%
WA	1618196	505282	311415	187811	98608	36858	1139974	41%
WI	1580612	799252	484481	57001	12214	5011	1357959	46%
WV	398316	273629	66252	21885	14322	6451	382539	49%
WY	52626	47978	46474	42696	35289	6902	179339	77%

Note: The "Zero Fast" column indicates the count of households that among the five nearest stations, there are no DC fast charging stations available. The "One fast" column represents the count of households that have only one DC fast charging station among the five nearest stations. The "Two fast stations" column reflects the count of households with two DC fast charging stations among the five nearest stations. The “Total number of households that have at least one fast charging station among the five nearest stations” columns are the sum of columns of “One fast station”, “Two fast stations”, “Three fast stations”, “Four fast stations”, and “Five fast stations”. The "Ratio of households with DC fast charging" column is computed by dividing the "Total number of households that have at least one fast charging station among the five nearest stations" by the entire number of households. State name abbreviation note: Alabama (AL), Alaska (AK), Arizona (AZ), Arkansas (AR), California (CA), Colorado (CO), Connecticut (CT), Delaware (DE), Florida (FL), Georgia (GA), Hawaii (HI), Idaho (ID), Illinois (IL), Indiana (IN), Iowa (IA), Kansas (KS), Kentucky (KY), Louisiana (LA), Maine (ME), Maryland (MD), Massachusetts (MA), Michigan (MI), Minnesota (MN), Mississippi (MS), Missouri (MO), Montana (MT), Nebraska (NE), Nevada (NV), New Hampshire (NH), New Jersey (NJ), New Mexico (NM), New York (NY), North Carolina (NC), North Dakota (ND), Ohio (OH), Oklahoma (OK), Oregon (OR), Pennsylvania (PA), Rhode Island (RI), South Carolina (SC), South Dakota (SD), Tennessee (TN), Texas (TX), Utah (UT), Vermont (VT), Virginia (VA), Washington (WA), West Virginia (WV), Wisconsin (WI), Wyoming (WY), and the District of Columbia (DC).

Approach 2: We introduce an alternative index that captures users’ preferences for various types of charging stations. Our findings show it consistently aligns with the overarching trends observed in our core assessment.

To account for potential user preferences for DC fast charging stations compared to Level 2 chargers, we conducted a new analysis using an alternative index we introduce. Initially, we separated regular charging, typically Level 2 charging, from DC fast charging, and computed the shortest distances from household addresses to their respective Level 2 (D_s) and DC fast charging

(D_f) stations, respectively. Here, we combine these two shortest distances, D_s and D_f, into a unified indicator, assigning distinct weights to the α, using the algorithm:

Step 1: Calculate the distance from each resident's address to the nearest Level 2 charging station (D_s) and Fast DC charging station (D_f) separately, for each of the 121 million households in the United States;

Step 2: Compare each household's D_s and D_f, and derive the final accessibility index using the following heuristics:

Rule 1: If D_s<= Walking distance, & D_f <= walking distance*, accessibility = min $\{D_s, D_f\}$
Rule 2: If D_s>Walking distance, & D_f <= walking distance*, accessibility = min $\{D_s, D_f\}$
Rule 3: If D_s <= Walking distance, & D_f> walking distance*, accessibility = min $\{D_s, D_f\}$
Rule 4: If D_s>Walking distance, &D_f> walking distance*, accessibility = min $\{D_s, \alpha D_f\}$, where α <1, and α is in the range of (0.5,1)

* walking distance: we have chosen 0.7 mile as the walking distance, which is about 15 min, from the literature [1].

Our heuristics are predicated on the assumption that when residents have access to charging stations within walking distance from their homes, the difference in preference between slow and fast charging becomes minimal. Residents can leave their cars charging and easily return home to engage in other activities. When the stations are situated beyond walking distance, it becomes more challenging for individuals to arrange their activities during the charging period, thereby increasing the attractiveness of fast charging. However, accurately quantifying the preference for fast charging over Level 2 charging in this scenario presents a significant challenge. Consequently, we conduct a sensitivity analysis by multiplying the shortest distance to fast charging stations with a weight, denoted as α, and allow α to range from 0.5 to 1.

Depending on the specific value of α(s) we adopt, we can derive a range of EV charging accessibility indexes. When α equals 0, it signifies the exclusive selection of DC fast charging stations, with no consideration for Level 2 charging stations within walking distance. Conversely, when α equals 1, both Level 2 and DC fast charging stations are assigned equal weight (our core analysis). We conduct a sensitivity analysis to bound our findings.

We re-run our LOESS analysis and examine income and racial/ethnic disparities within the spectrum of EV accessibility using our index. Remarkably, our sensitivity analysis demonstrates consistent trends across the range of $\alpha's$ we tested (illustrated in Supplementary Figure 22). These results are with our core analytic results. Similarly, our examination of the geographical distribution of income and racial/ethnic disparities (as demonstrated in Supplementary Figure 23), even when employing the most aggressive weight α of 0.5, still yields results that closely mirror our findings outlined in the manuscript. Thus, our conclusions in the manuscript are robust.

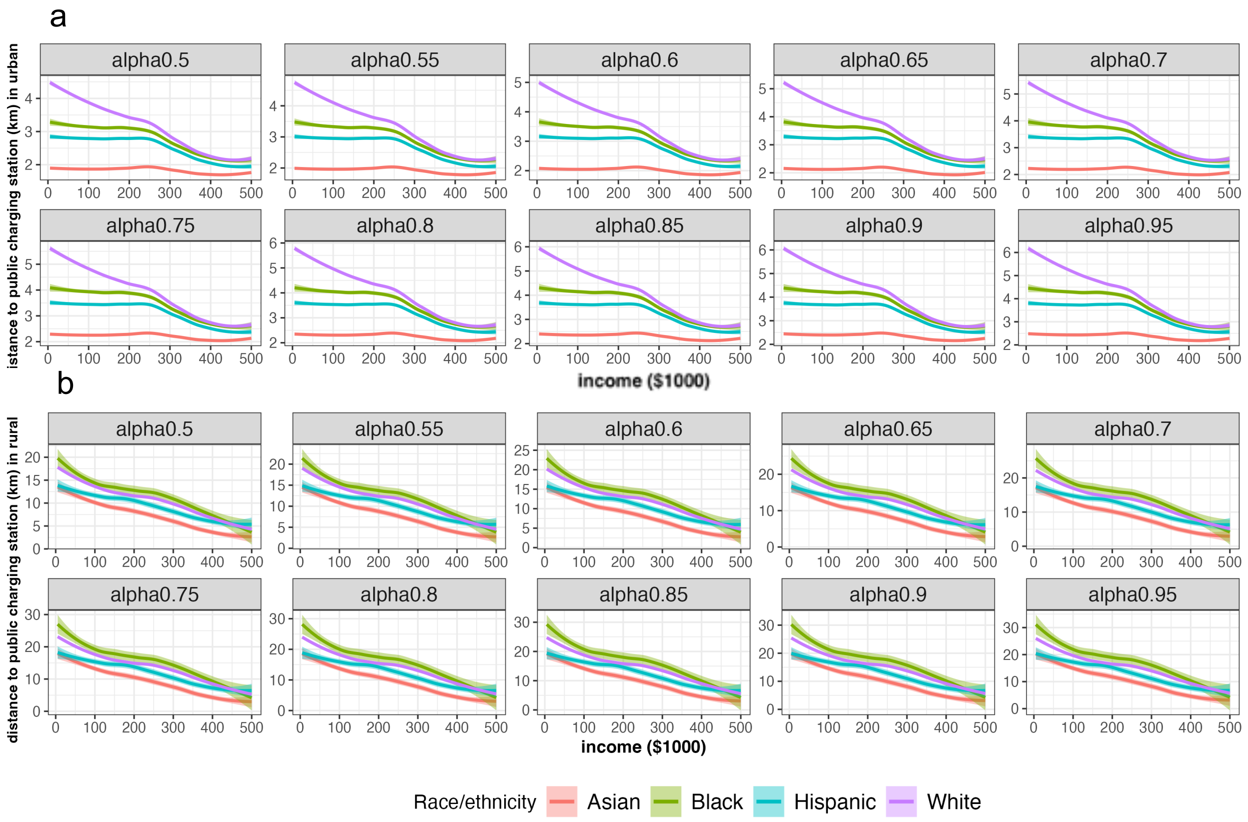

Supplementary Figure 22: Locally weighted scatterplot smoothing (LOESS) regression curves among different/ethnic populations by average distance to nearest electric vehicle (EV) infrastructure in rural and urban areas based on the weights we assigned to the fast-charging station. a. LOESS outcomes in urban setting by alphas (α) from 0.5 to 0.95. b. LOESS outcomes in rural setting by alphas from 0.5 to 0.95. “alpha0.5” means we assign 0.5 weight to fast charging, whereas “alpha0.95” means we assign a weight of 0.95, and so forth. The top panel of ten subplots represents results for urban households, while the bottom panel of ten subplots represents results for rural households. Our sensitivity analysis demonstrates consistent trends across the range of $\alpha's$ we tested. These results are with our core analytic results.

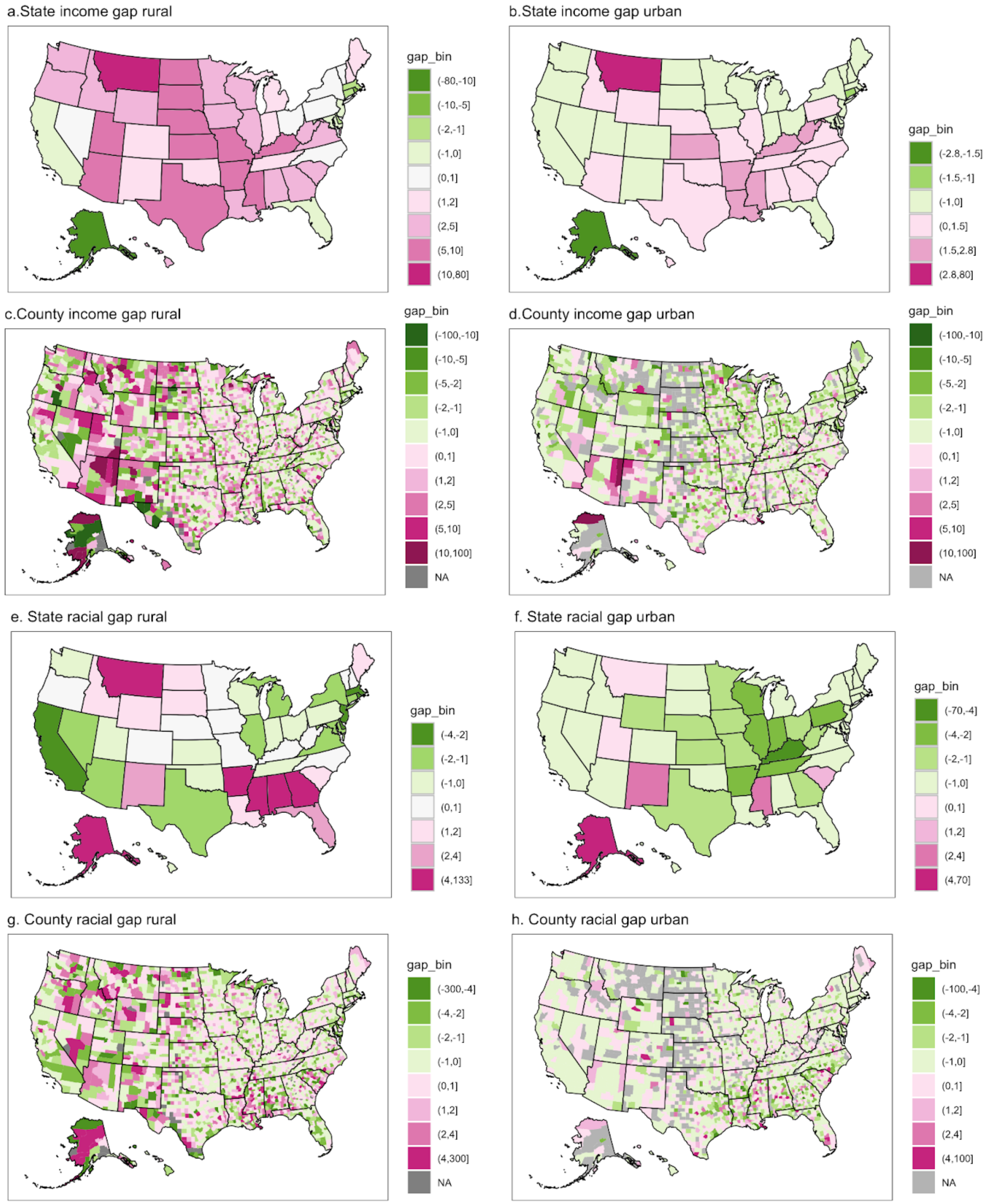

Supplementary Figure 23: Equity assessment through accessibility gaps at state and county level using alpha (α) 0.5. a-d: accessibility gaps between low-moderate income (LMI) and non-LMI households by their location at state and county; e-h: accessibility gaps between Black and White households by their location at state and county. The magenta color scheme shows more

greater gaps, and the green color scheme shows less gaps. Gray color means no data available or beyond the defined minimum and maximum thresholds of -100 and 100, respectively.

Approach 3: We conduct the analysis using the Inhabitant-to-Station Ratio as suggested by a reviewer.

To strike a balance between representing income and racial distributions while ensuring access to sufficient EV charging infrastructure, we apply a two-tiered approach to calculate the Inhabitant-to-Station Ratio (ISR) at the zip code and the census tract levels.

1. At the zip code level, our choice is rooted in the fact that our rural/urban categorization, based on U.S. Census data, operates at this level. Consequently, we can segregate zip codes into rural and urban categories and calculate the ISR accordingly. To classify zip codes into LMI (Low to Moderate Income) and non-LMI groups, as well as Black and White demographic categories, we followed these steps: We utilize median household income at the county level as the threshold. If the median household income at the zip code level falls below this threshold, we categorize it as LMI; if it surpasses the threshold, it is labeled as non-LMI. For racial/ethnic categorization, if the percentage of the Black population exceeds the percentage of any single race/ethnicity (including White, Hispanic, Asian, Hawaiian, Native American, etc.), the zip code is classified as Black-dominant, and vice versa for White-dominant zip codes. Subsequently, we computed the ISR gaps between LMI and non-LMI and Black and White households, aggregating the means of ISRs at the state level, mirroring the format presented in Figure 2 in our manuscript. Please refer to Supplemental Figure 24 for the ISR at the state level based on zip code information.

2. Moving to the census tract level, these areas are smaller than zip codes and represent the lowest jurisdictional level for which we have comprehensive data, including the number of households, EV charging stations, racial/ethnic demographics, median household income, and more. We applied a similar methodology to our zip code approach to calculate the ISR gap, with one additional step of assigning a rural/urban indicator to each tract. This step is necessary since rural/urban classification is only available at the zip code level. We use a 2010 ZCTA to Census Tract Relationship File to link zip codes to census tracts. In cases where a tract spanned multiple zip codes, we distributed the tract's population among the relevant zip codes and linked it to the one with the largest population share. Please refer to Supplemental Figure 25 for the ISR at the state level based on census tract information.

There are some concerns regarding the utilization of the IRS that we note. First, it is essential to acknowledge that there will be varying outcomes in IRS data across the two levels of study we have adopted. Second, we lack information for certain states when employing IRS methods; this issue becomes particularly prominent in the analysis of racial disparities. This is primarily attributable to the inherent trade-off between granularity and aggregation. 1) When opting for the tract level, a significant number of tracts without public EV charging stations end up being excluded. As a result, we are left with only 16,203 tracts out of 85,187. Subsequently, when we aggregate our analysis to the state level, some states, particularly those in rural areas, may not

have enough either LMI or non-LMI tracts to facilitate a meaningful comparison. In fact, in certain cases, we may be forced to exclude an entire state (e.g., state Louisiana) for the income gap analysis, and most of the states in the racial gap analysis, because there are states that do not have Black-dominant tracts. 2) If we decide to use zip code data instead, we risk losing the ability to capture the intricate dynamics of income and racial distribution due to the broader geographical scale of zip codes compared to tracts. This issue becomes more pronounced in the context of racial analysis. The point of this is that we expect our original results derived using the individual household level will yield more robust results when contrasted with higher-level aggregation analyses. Nonetheless, for those geographies that are available, we find consistent results to our base analysis.

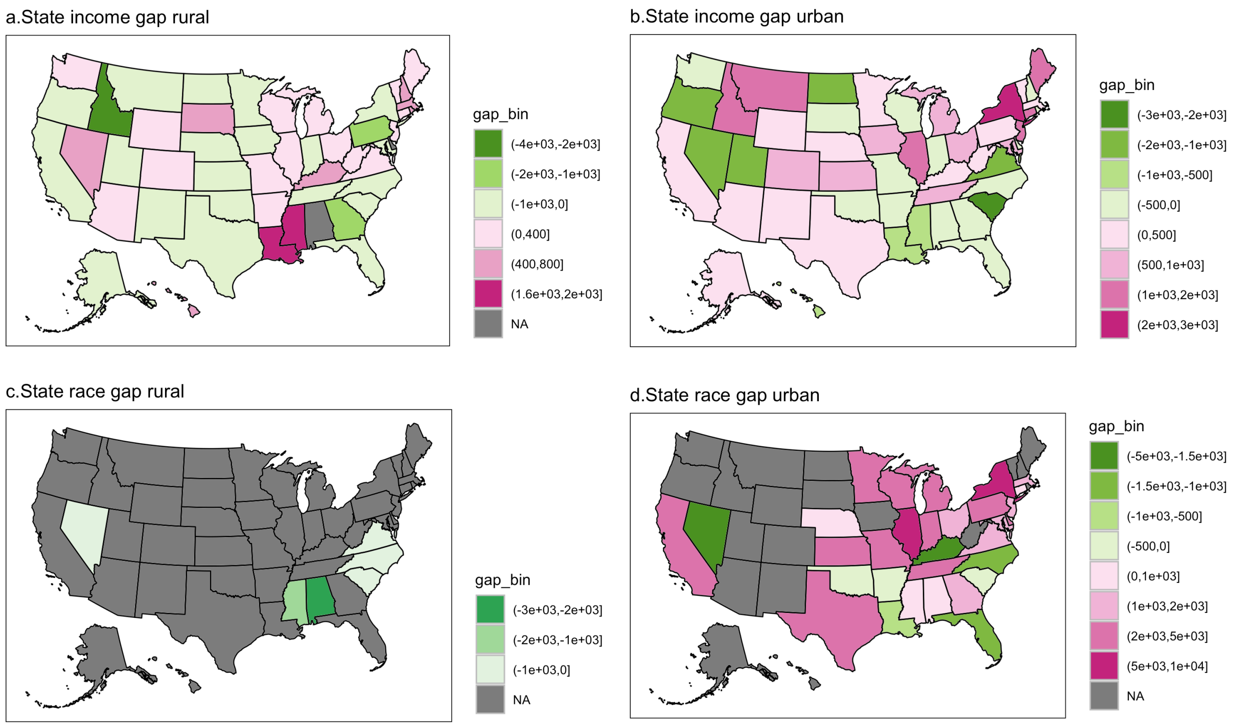

Supplementary Figure 24. Equity assessment through Inhabitant-to-Station Ratio (ISR) gaps at state level based on the zip code. a and b: ISR gaps between low-moderate income (LMI) and non-LMI households by their location at state level; c and d: ISR gaps between Black and White households by their location at state level. The magenta color scheme shows LMI and Black households experienced larger ISR gaps (or barriers) compared to non-LMI and White households, respectively. Vice versa, the green color suggests the opposite. Gray color means no data available.

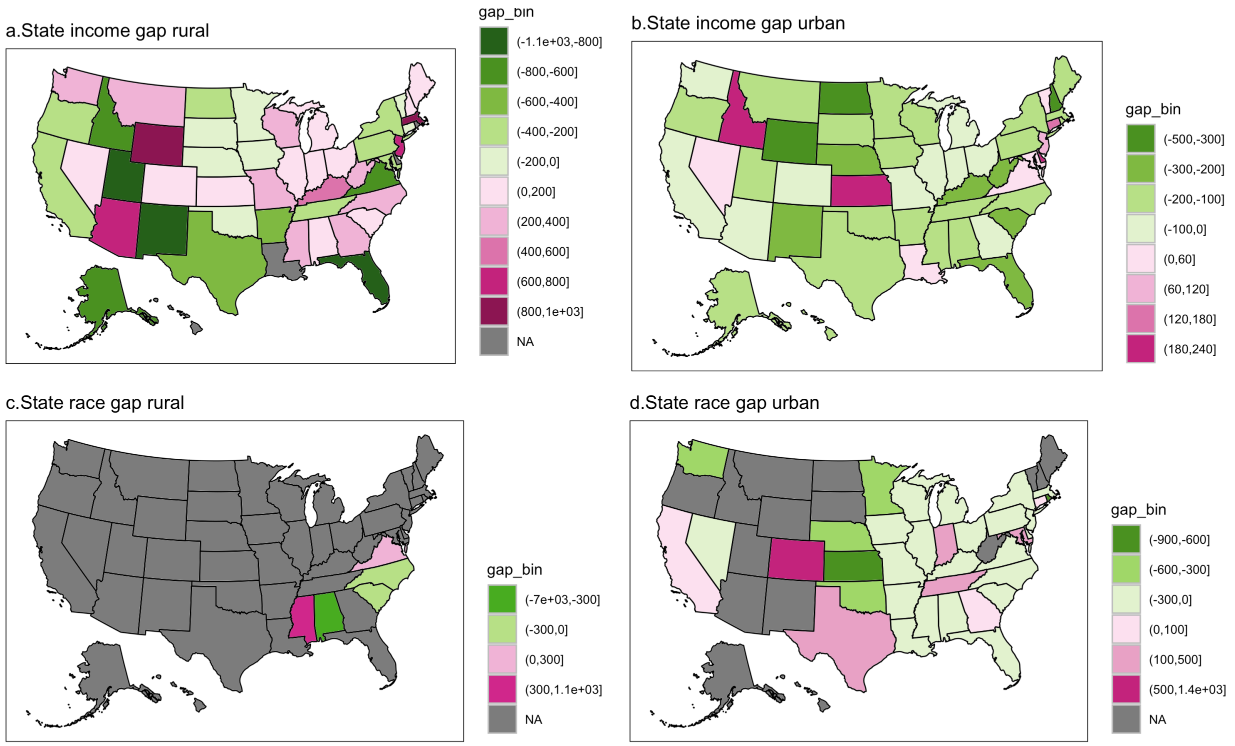

Supplementary Figure 25. Equity assessment through Inhabitant-to-Station Ratio (ISR) gaps at state level based on the census tract. a and b: ISR gaps between low-moderate income (LMI) and non-LMI households by their location at state level; c and d: ISR gaps between Black and White households by their location at state level. The magenta color scheme shows LMI and Black households experienced larger ISR gaps (or barriers) compared to non-LMI and White households, respectively. Vice versa, the green color suggests the opposite. Gray color means no data available.

Supplementary Note 2

This supplementary note introduces an additional dimension to the accessibility gap by directly incorporating the concept of Disadvantaged Communities (DAC) as defined by the Electric Vehicle (EV) Charging Justice40 Map Tool.

The EV Charging Justice40 Map Tool, a collaborative effort between the Department of Energy and the Department of Transportation, encompasses 36 and 22 indicators that define Disadvantaged Communities (DAC) at the census tract level (see Supplementary Figure 26). This tool offers a comprehensive perspective on DACs. To integrate this dimension into our analysis, we assigned each household a binary variable denoting its DAC status (1 for DAC, 0 for non-DAC). Subsequently, we calculated the accessibility gap between DAC and non-DAC households at both county and state levels.

This expanded analysis allowed us to explore correlations between our racial accessibility gap and the DAC accessibility gap, as well as between our income accessibility gap and the DAC accessibility gap, both within counties and across states. For detailed correlation results, please refer to Supplemental Table 9. We highlight a significant limitation associated with the use of the DAC classification. We've noted that many counties are categorized as either entirely non-DAC or entirely DAC, despite the DAC classification being based on the census tract level. Consequently, this results in a significant reduction in the number of counties included in our final county-level correlation analysis. To provide context, there are 860 counties that do not contain any DAC census tracts, and 273 counties exclusively consist of DAC census tracts. In such cases, where counties lack diversity in their DAC status, we encounter limitations in our ability to compare accessibility gaps between DAC and non-DAC groups within these counties. This phenomenon contributes to the observed decrease in the strength of correlation at the county level compared to the state level.

Supplementary Table 9. Correlations Between the Racial Accessibility Gap and the Disadvantaged Communities (DAC) Accessibility Gap, and Income Accessibility Gap and the DAC Accessibility Gap.

Correlation	Income accessibility gap and DAC accessibility gap	Racial accessibility gap and DAC accessibility gap
**State**	0.43	0.27
**County**	0.31	0.21

It's crucial to underscore that our dataset operates at the household level, affording us the capability to delve into fine-grained details regarding income and race. Any level of aggregation above household will necessarily be less rigorous. Other metrics, e.g., "Inhabitant-to-Station Ratio" do not capture individual-level variation. Our study's granularity (i.e., the household) instills confidence in the robustness of our results when juxtaposed with the DAC accessibility gap. We

note that our analysis suggest that the Justice40 tool may be too blunt to capture important variations in EV infrastructure access.

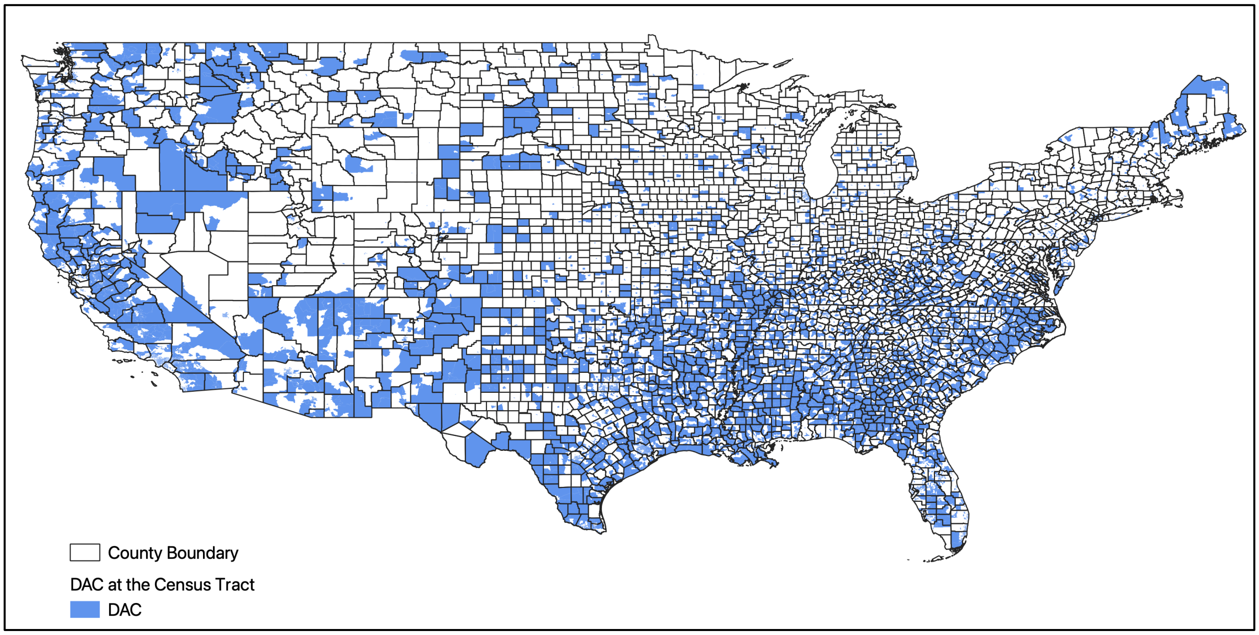

Supplementary Figure 26. Department of Energy (DOE)/Department of Transportation (DOT)'s interim definition for disadvantaged communities (DACs). This map depicts the DACs at the census tract level from 36 and 22 indicators.

Supplementary Note 3. EV Charging Regulations and Policies

The current federal and some state policies related to EV public infrastructure emphasize increasing the availability of charging stations and providing incentives for their installation. However, state's that have policies mostly focus on broader installation, with a few states placing equity considerations front and center. It is crucial to navigate the development of EV charging stations in disadvantaged regions, ensuring that the benefits of electric transportation are accessible to all members of society. We conducted a comprehensive analysis of EV charging regulations and incentives from the federal and state government. We found that there are only 26 existing regulations aiming to promote equitable access to electric vehicle charging infrastructure implemented in 16 states. In contrast, incentives are more common in states across the US. It is evident that the United States currently lacks well-defined regulations and rules to promote equitable access to electric vehicle charging infrastructure [2]. This indicated a clear gap of regulations from the state government which suggest that subnational government should implement tailored solution.

Regulations/Rules

At the federal level, one of the most significant motivators of equity and access within EV charging development is the Justice40 program, which sets a goal of providing 40% of the benefits of relevant programs to disadvantaged communities (DACs). A major federal program under the umbrella of Justice40 is the NEVI Formula Program, which requires states to submit comprehensive plans in order to receive crucial funding for deploying EV charging around important highways [3]. In its administration of this program, the federal government has both directly and indirectly prioritized equity concerns. First, states are required to touch on equity in their NEVI plans, although there is an absence of specific requirements in terms of implementation. The Federal Highway Administration implemented NEVI standards and requirements to ensure federally funded charging stations meet a certain level of accessibility, including charging network connectivity and publicly available price and availability information [4].

At the state level, there is significant variation in implementation of NEVI guidance as well as local EV infrastructure policy [3]. To start, states have taken an array of strategies to plan for Justice40 compliance. Most states that submitted NEVI plans use the Electric Vehicle Charging Justice40 Map created from the Department of Transportation and Department of Energy's joint interim definition of DACs [3,5]. In addition, a number of states have integrated tools that utilize more specific data available to states that may not be consistent enough across the country to be used in the federal tool. These generally utilize multiple data sets to measure the climate and pollution burdens faced by each census tract or locality, considering factors like air quality, health risks, and socioeconomic demographics. States also do not have a consistent definition of what a "benefit" looks like to a DAC, and to some degree have used their NEVI plans to describe these benefits with varying degrees of specificity. State defined benefits have included air quality

benefits most prevalently, as well as workforce participation and economic development from tourism [3]. Finally, some states conducted community outreach to formulate their NEVI plans. Successful approaches ranged from social media campaigns and targeted newspaper ads to regional workshops. However, one report states these activities “were lacking involvement from groups that represented low-income communities, communities of color, and environmental justice communities” [3]. While most states did engage with the public, some only held private meetings; states also varied in the degree to which they disclosed the specifics of their outreach and incorporated feedback in NEVI plan formulation [3].

Outside of NEVI, there has been little coordination at the state level to facilitate equitable EV regulations. Of the regulations that are in place, most are at best indirectly linked to equity and access. In our database, we are only able to find 16 states implemented regulations to ensure the equitable accessibility, see supplementary Table 10.

Supplementary Table 10 A List of Regulations Among 16 States.

State	Policy Title	Law referred to	Policy related to equity	Targeted Communities	Date Started	Links/Documents
**California**	Electric Vehicle (EV) Charging Station Assessment	California Public Resources Code 25229	In addition, the assessment must analyze the existing and future infrastructure needs across California, including in **low-income communities**.	Low-income		http://leginfo legislature.ca.gov/
**California**	Electric Vehicle (EV) Charging Station Uptime Reporting Standards	Assembly Bill 2061, 2022	The assessment must include considerations for equitable access to EV charging stations in **low-,** moderate-, and high-**income communities.**	Low-income	2022	https://leginfo.legislature.ca.gov/faces/home.xhtml
**California**	Zero Electric Vehicle (ZEV) Office Authorization and Equity Assessment	Senate Bill 1251, 2022	1. Improve access to ZEVs, supporting infrastructure, and ZEV transportation options in **low-income, disadvantaged, and underserved communities**. 2. Reduce pollution from transportation in **low-income, disadvantaged, and underserved communities**.	low-income, disadvantaged, and underserved communities	2022	https://leginfo.legislature.ca.gov/faces/billNavClient.xhtml?bill_id=202120220SB1251
**California**	Zero Emission Vehicle (ZEV) Initiative	California Health and Safety Code 44258.4	1. In consultation with the State Energy Resources Conservation and Development Commission, CARB prepared a funding plan (PDF) that includes a market and technology assessment, assessments of existing zero and near-zero emission funding programs, and programs that increase access to **disadvantaged, low-income, and moderate-income communities** and consumers. 2. Potential programs under the initiative include those involving innovative financing, car sharing, charging infrastructure in **multi-unit dwellings located in disadvantaged communities**, public transit, and agricultural vanpool programs.	Disadvantaged, low-income, moderate-income communities, and Multiunit Dwellings		http://www.oal.ca.gov/
**California**	Zero Emission Vehicle (ZEV) Promotion Plan	Executive Order B-16, 2012, Executive Order B-48, 2018, and Executive Orders N-19-19, 2019	Update the 2016 ZEV Action plan, with a focus on **low income and disadvantaged communities**.	Low income and disadvantaged communities	2012	https://www.gov.ca.gov/category/executive-orders/
**California**	Electric Vehicle (EV) Pilot Programs	Public Utilities Code 740.13-740.14	Priority must be given to locations in **disadvantaged communities**, as defined by the California Environmental Protection Agency.	Disadvantaged communities	Sep-20	https://vacleancities.org/mid-atlantic-electrification-partnership/
Hawaii	Electric Vehicle (EV) Charging Station Rebate Program Authorization	Hawaii Revised Statutes 243-3.5, 269-72 and 269-73	The PUC must prioritize rebate awards for EV charging stations that are publicly available; serve fuel cell electric vehicle fleets; serve multiple tenants, employees, or customers; support tourism; or serve **low- or moderate-income or environmental justice communities**.	Low- or moderate-income or environmental justice communities		https://www.capitol.hawaii.gov/
Illinois	Electric Vehicle (EV) Charging Station Building Standards for Residential Developments	Senate Bill 40, 2023	1. New single-family homes or small multifamily dwellings that qualify as **affordable housing** must have one EV-capable parking space per dwelling. 2. Building permits for **affordable housing** single-family and multifamily developments must meet the following benchmarks to support the development of EV-capable parking spaces.	Affordable Housing	2023	https://www.ilga.gov/legislation/billstatus.asp?DocNum=0040&GAID=17&GA=103&DocTypeID=SB&LegID=142908&SessionID=112
Illinois	Regional Electric Vehicle (REV) Midwest Plan	REV Midwest Partnership Announcement	Identify historically **disadvantaged communities** for equitable EV charging station development and EV adoption.	Disadvantaged communities		https://www.michigan.gov/whitmer/0,9309,7-387-90499_90640-569470--,00.html
Indiana	Regional Electric Vehicle (REV) Midwest Plan	REV Midwest Partnership Announcement	Identify historically **disadvantaged communities** for equitable EV charging station development and EV adoption.	Disadvantaged communities		https://www.michigan.gov/whitmer/0,9309,7-387-90499_90640-569470--,00.html

Indiana	Utility Electric Vehicle (EV) Charging Station Pilot Program Authorization	Indiana Code 8-1-43	Utilities must also include plans to install EV charging stations in **underserved and diverse communities**.	Underserved and Diverse communities		http://www.in.gov/legislative/ic/code/
Maryland	Utility Electric Vehicle (EV) Charging Station Deployment Authorization and Requirements	House Bill 834, 2023, Public Service Commission Order 88997 – Case No. 9478, and Maryland Statutes Public Utilities Code 7-901 through 7-905	Beginning October 1, 2023, IOUs that participate in the PSC EV Pilot Program may install Level 2 EV charging stations at **multi-unit dwellings in underserved communities**.	Underserved Communities	2023	https://vacleancities.org/mid-atlantic-electrification-partnership/
Maryland	Mid-Atlantic Region Electric Vehicle (EV) Support	MAEP Website	Participating States commit to creating a regional network of EVs and EV charging stations that will make it possible to seamlessly operate light-, medium-, and heavy-duty EVs across transportation corridors and in **low-income communities**.	Low-income	Sep-20	https://mgaleg.maryland.gov/mgawebsite/Legislation/Details/hb0834?ys=2023RS
Massachusetts	Electric Vehicle (EV) Charging Infrastructure Deployment Support	Session Law Chapter 179, Section 81, 2022	Opportunities for EV charging stations in **urban, suburban, rural, and low- and moderate-income areas**.	Low- and moderate-income areas, and Rural	2022	https://malegislature.gov/Bills/192/H5060
Michigon	Regional Electric Vehicle (REV) Midwest Plan	REV Midwest Partnership Announcement	Identify historically **disadvantaged communities** for equitable EV charging station development and EV adoption.	Disadvantaged communities		https://www.michigan.gov/whitmer/0,9309,7-387-90499_90640-569470--,00.html
Minnesota	Regional Electric Vehicle (REV) Midwest Plan	REV Midwest Partnership Announcement	Identify historically **disadvantaged communities** for equitable EV charging station development and EV adoption.	Disadvantaged communities		https://www.michigan.gov/whitmer/0,9309,7-387-90499_90640-569470--,00.html
New York	Zero Emission Vehicle (ZEV) Requirements	Senate Bill 7788, 2022	Strategies to accelerate deployment of affordable ZEV infrastructure that serves **low-income and disadvantaged communities**.	Low-income and disadvantaged communities	2022	https://nyassembly.gov/leg/?bn=S07788&term=2021
Oregon	Volkswagen (VW) Settlement Allocation	Executive Order 17-21, 2017	The plan (PDF) includes the development and maintenance of electric vehicle charging stations, with a focus on **rural and low-income communities**.	Rural and low-income communities	2017	http://www.oregon.gov/gov/admin/pages/executive-orders.aspx
Vermont	Utility Electric Vehicle (EV) Program Funding Requirement	Senate Bill 99, 2023	IOUs must prioritize **low-income communities** in their incentive programs.	Low-income communities	2023	https://legislature.vermont.gov/bill/status/2024/S.99
Vermont	Electric Vehicle (EV) Charging Station Multi-Unit Dwelling (MUD) Pilot Program	Act 55, 2021	The Vermont Agency of Transportation must establish and administer, through a memorandum of understanding with the Department of Housing and Community Development (DHCD), a pilot program to support the installation of EV charging station at MUDs and **affordable housing units**.	Affordable housing units		https://legislature.vermont.gov/
Virginia	Mid-Atlantic Region Electric Vehicle (EV) Support	MAEP Website	Participating States commit to creating a regional network of EVs and EV charging stations that will make it possible to seamlessly operate light-, medium-, and heavy-duty EVs across transportation corridors and in **low-income communities**.	Low-income	Sep-20	https://vacleancities.org/mid-atlantic-electrification-partnership/
Virginia	Transportation Electrification Study	House Bill 2282, 2021	Utility and public investments that complement private efforts to deploy electric vehicle (EV) charging stations, focusing on **low-income, minority, and rural communities**.	Low-income, minority, and rural communities	2021	https://lis.virginia.gov/
West Virginia	Mid-Atlantic Region Electric Vehicle (EV) Support	MAEP Website	Participating States commit to creating a regional network of EVs and EV charging stations that will make it possible to seamlessly operate light-, medium-, and heavy-duty EVs across transportation corridors and in **low-income communities**.	Low-income	Sep-20	https://vacleancities.org/mid-atlantic-electrification-partnership/
Wisconsin	Regional Electric Vehicle (REV) Midwest Plan	REV Midwest Partnership Announcement	Identify historically **disadvantaged communities** for equitable EV charging station development and EV adoption.	Disadvantaged communities		https://www.michigan.gov/whitmer/0,9309,7-387-90499_90640-569470--,00.html

State incentives

Under state incentives, a diverse array of instruments and tools are employed to promote environmental awareness encompassing tax credits, financial incentives, rebates, prepaid charge cards, both public and residential EV chargers, memberships for publicly-owned stations, educational programs, enhancements to the electric distribution system infrastructure, and numerous other strategies. These kinds of programs can be divided into 5 major categories - Tax Credit, Rebate, Public EV Charging Stations, Funds, and Educational Programs.

Sustainable Building Tax Credit (SBTC) [6] in New Mexico offers tax credit for installation of energy-efficient infrastructure to low-income residents. Programs offering rebates to incentivize the purchase and installation of Level 2/direct current fast charging (DCFC) stations or utility-owned charging stations in multi-family housing, and disadvantaged or low-income communities, include the EV Make-Ready Program [7] in New York, Commercial Electric Vehicle Charging Station Rebate by United Illuminating [8](UI) in Connecticut, Residential EV Charger Rebate [9] in Burbank, Plugging In Oregon Community Charging Rebates [10] in Oregon, Electric Vehicle Charging Pilot Program [11] in Maryland Green New Deal [12] and Charge Up LA! Residential [13] in Los Angeles, Residential EV Charging Incentive Program [14] in Diamond Bar, and a range of programs such as Golden State Priority Project [15], Small Site Rebate , New Construction Rebate [16], Charging Infrastructure and Rebate [17], and Empower EV Program [18] in California. Clean Cars 4 All [19] is another rebate program in California that also provides pre-paid charge cards. County including Cook County, and major states and cities like Maryland, New York, Dallas, and Seattle, have implemented a range of programs like Electric Vehicle Charging Stations Program [20], Electric Vehicle Charging Pilot Program [11], PlaNYC: Getting Sustainability Done [21], Comprehensive Environmental and Climate Action Plan [22], and Seattle City Light [23] share the common objective of installing public EV charging stations in under-resourced and vulnerable community areas. Programs like Electric Vehicle Supply Equipment (EVSE) Grant Program [24] in Vermont, Turn-Key Installation [25] in California, and It Pay$ to Plug In [26] and Electric Vehicle and EV Charging Station Grant Program [27] in New Jersey provide grants to design and install EV charging equipment. Furthermore, they fund programs to modify existing EV charging station grant programs. The state of Oregon offers a program - EV Charging Infrastructure Education [28] which provides a self-guided course covering essential topics required for maximizing federal charging investments and establishing effective charging programs in underserved and rural communities. Another campaign called Electric For All [29] in California provides information on charging station incentives, beneficial time-of-use rates for EV charging, and reduced vehicle registration fees and high occupancy lane exemptions by offering a zip-code based “incentives and perks” search tool with income-based eligibility requirements.

Supplementary Note 4. A systematic literature review for EV accessibility and its contributing factors

Using the search protocol presented in Petticrew and Roberts 2008 [30], we conducted a comprehensive literature review, including both peer-reviewed journals and conference papers.

The search protocol was implemented using Web of Science to identify potential papers and designed capture the maximum number of papers related to electric vehicle infrastructure accessibility, with a specific focus on equity. The search terms used were (EV infrastructure OR EV charger) AND (disparity OR disparities OR inequitable OR equity OR equitable). Systematic literature reviews necessitate the explicit application of criteria to filter and include specific references. Here, we include papers if they were academic peer-reviewed, conference papers, or dissertations. Papers were excluded if they were published more than five years ago or focused on private EV infrastructure.

We identified 17 references for this review. In Supplementary Table 11, we provide an overview of how we arrived at the final set of papers and the distribution of references within our review sample.

Supplementary Table 11. Total Number of Papers Included in This Literature Review

Key words	Outcome
EV infrastructure AND disparit*	23
EV infrastructure AND equit*	37
EV infrastructure AND inequitable	3
EV charger AND disparit*	6
EV charger AND equit*	4
EV charger AND inequitable	0
EV charging infrastructure AND disparit*	19
EV charging infrastructure AND equit*	12
EV charging infrastructure AND inequitable	3
**Total**	**107**
Duplicate	46
Unrelated and not specifically mentioned factors	41
Private EV infrastructure	3
**Final number**	**17**

Note: EV means electric vehicle.

We evaluated the 17 papers in our sample to assess the factors that will impact the accessibility of EV infrastructure. These factors are illustrated in Supplementary Table 12.

Supplementary Table 12. A Full List of the 17 Papers.

Papers	Methods	Level of Study	Coverage	Factors
31	Machine learning	census block group	Orange County, California	Vehicles Available<br>Population Density<br>Poverty<br>Education<br>Income<br>Household size<br>Housing affordability<br>Average commute time<br>Age between 25 to 45 (%)<br>Employment Rate<br>PM 2.5 level<br>Traffic impact
32	Generalized additive model	census block group	California	Distance to nearest highway or freeway<br>Multi-unit housing unit rate<br>Median household income
33	Spatial autocorrelation	city level (top 10 cities)	China	GDP<br>Population<br>Area
34	A hierarchical agglomerative clustering method	trip diary data	USA	Income
35	Literature Review	Articles	All	Age<br>Income<br>Mobility
36	Correlation analyses	Zipcode	New York City	Median household income<br>Race (%)<br>Presence of highways<br>Poverty rate
37	A self-scheduling model		Los Angeles	Income
38		census tracts	Seattle, Washington	Income<br>Homeowner rate
39	Descriptive		USA	Income<br>Population density
40	Logit	household	Ireland	Income
41	Equitable placement model	Chargers	San Francisco	Race<br>Income<br>Culture<br>Education
42	Optimization model	vehicles	USA	Rural/Urban<br>Income
43	a positive review and value argumentation method		Dundee City, UK	Income<br>Employment<br>Crime and health deprivation<br>Disability.
44	Spatial analysis	permit, tract-level data	Seattle, WA	Gini<br>Poverty<br>HomeValue<br>Income<br>White

				Education<br>SingleFamily<br>HomeOwn<br>Population density
45	EV-user-centric Deployment Decision-making Model	census tracts	Los Angeles County	Gini
46	Descriptive	Descriptive	All	Rural/Urban<br>Income
47	Descriptive	county level	USA	Rural/Urban

The table offers valuable insights into the key factors discussed within the selected references. Notably, “income” emerges as the most frequently mentioned factor, indicating that researchers often emphasize the role of income levels in discussions related to electric vehicle infrastructure accessibility. This underscores the significance of economic considerations in assessing and addressing disparities in this context. Following closely behind income, “population density” stands out as another prominent factor. It suggests that the population density of an area is a critical determinant of EV infrastructure accessibility. The “rural/urban” divide also appears as a notable consideration, highlighting that the urban-rural distinction is relevant in discussions regarding EV infrastructure. “distance to nearest highway or freeway” is another factor of significance. This factor emphasizes the role of transportation infrastructure in ensuring convenient access to EV charging. The “multi-unit housing unit rate” factor suggests that the type of housing people reside in can influence their ability to access EV charging. Education and other factors also make appearances, indicating the multidimensional nature of the issue.

In summary, the table reveals a spectrum of factors that researchers have identified as relevant in the discussion of EV infrastructure accessibility and equity. These factors encompass economic, demographic, geographical, and housing-related considerations, underlining the complexity of the issue and the need for a holistic approach to achieving equity in electric vehicle infrastructure.

Building upon the insights gained from the literature review, we run regression analyzes to empirically test the influences of these key factors on the EV infrastructure accessibility gap. Our regression results show the significance of the highway gap as a primary contributor to the accessibility gap, closely followed by the income gap. Furthermore, our observations indicate that MUD (Multi-Unit Dwelling) gaps also wield a crucial influence in contributing to the accessibility gap between rural and urban settings. This can be attributed to the elevated presence of charging stations around MUDs in urban areas.

Supplementary Note 5. Adopted Terminology for Describing Our Research Groups

To ensure that our manuscript maintains unbiased language when referring to racial and ethnic populations, we have adhered to the guidelines provided by the American Psychological Association (APA) on Racial and Ethnic Identity.[48].

1. In the manuscript, when addressing "racial/ethnic households" or "racial/ethnic populations" we specifically refer to four groups: Black, White, Hispanic, and Asian households, as outlined in the manuscript. The term "racial disparity" in our study specifically pertain to Black and White households, as noted in our manuscript.
2. When describing the DataAxle dataset, we use the term "racial/ethnic households" or "racial/ethnic populations" as the data provide detailed information that allows us to categorize households by four distinct racial/ethnic categories.
3. In our first section "Nation-wide social disparity in access to public EV infrastructure", We employ the term "racial/ethnic" to encompass the entirety of our sample population, as well as in reference to the initial research design aimed at delineating the nonlinear relationship between income and accessibility across racial/ethnic households. Within this context, we distinguish four primary groups of households. Hence, "racial/ethnic households" is exclusively utilized when discussing the entire sample.
4. In the following sections, which form the core of our analysis, the term "racial disparity" is specifically employed due to its essential role in our research. The subsequent research designs (second to fourth) aim to evaluate the racial disparity between White and Black households by examining the discrepancies in their EV infrastructure accessibility. Consequently, "racial" or "racial disparity" is utilized in these sections.
5. As the core analysis focuses on comparing White and Black households, our paper's title is "Income and **Racial Disparity** in Household Publicly Available Electric Vehicle Infrastructure Accessibility."
6. The term "underrepresented groups" refers to non-White racial/ethnic populations, which comprise a smaller proportion of key demographic subgroups compared to their White counterparts in the general population. In our manuscript, we primarily use "underrepresented groups" or "underrepresented households." However, we retain the term "people of color" when specifically referencing literature that employs this terminology.

Supplementary References

1. Yang, Y. & Diez-Roux, A. V. Walking Distance by Trip Purpose and Population Subgroups. *American Journal of Preventive Medicine* **43**, 11–19 (2012).
2. Smart Cities Dive. EV charging station rollout hampered by outdated state, city regulations: report. *Smart Cities Dive* https://www.smartcitiesdive.com/news/ev-charging-stations-hampered-outdated-state-city-policies-regulations-permits/634819/ (2022).
3. Patterson, R. *et al.* How States Can Lead on Racial and Economic Equity through the National Electric Vehicle Infrastructure (NEVI) Program. (2023).
4. National Electric Vehicle Infrastructure Standards and Requirements. *Federal Register* https://www.federalregister.gov/documents/2023/02/28/2023-03500/national-electric-vehicle-infrastructure-standards-and-requirements (2023).
5. Lyons, T. Incorporating Equity and Justice40 in NEVI and Beyond. (2022).
6. Energy, Minerals and Natural Resources Department. Sustainable Building Tax Credit (SBTC). *Energy Conservation and Management* https://www.emnrd.nm.gov/ecmd/tax-incentives/sustainable-building-tax-credit-sbtc/ (2021).
7. Joint Utilities of New York. EV Make-Ready Program. https://jointutilitiesofny.org/ev/make-ready (2020).
8. Avangrid. Commercial Electric Vehicle Charging Station Rebate by United Illuminating (UI). *uinet* https://www.uinet.com/w/united-illuminating-calls-for-contractors-for-statewide-electric-vehicle-charging-program?p_l_back_url=%2Fsearch%3Fq%3DCommercial%2520Electric%2520Vehicle%2520%28EV%29%2520Charging%2520Station%2520Rebate (2022).

9. Burbank Water & Power. Residential EV Charging Stations Rebate. *Burbank Water & Power* https://www.burbankwaterandpower.com/electric-vehicles/residential-ev-charger-rebate (2019).
10. Forth. Plugging In Oregon Community Charging Rebates. https://forthmobility.org/Community Charging Rebate (2023).
11. Maryland Public Service Commission. Electric Vehicle (EV) Charging Pilot Program. *Maryland Public Service Commission* https://www.psc.state.md.us/wp-content/uploads/PC44-EV-Pilot-Fact-Sheet-4.pdf (2023).
12. City of Los Angeles. *L.A.'s Green New Deal*. (2019).
13. Los Angeles Department of Water and Power. Charge Up LA! Residential. https://www.ladwp.com/ladwp/faces/wcnav_externalId/r-sm-rp-ev?_afrWindowId=null&_afrLoop=1128087689503446&_afrWindowMode=0&_adf.ctrl-state=ux4vaz2u9_4#%40%3F_afrWindowId%3Dnull%26_afrLoop%3D1128087689503446%26_afrWindowMode%3D0%26_adf.ctrl-state%3Dhch3nr8ln_4 (2013).
14. South Coast Air Quality Management District. Residential EV Charging Incentive Program. https://www.aqmd.gov/home/programs/community/community-detail?title=ev-charging-incentive (2023).
15. California Energy Commission. Golden State Priority Project. https://calevip.org/incentive-project/gspp-incentive-east-central (2023).
16. Southern California Edison. New Construction Rebate Program. https://www.sce.com/evbusiness/chargeready/new-construction-rebate (2023).
17. Southern California Edison. Charging Infrastructure and Rebate Program. https://www.sce.com/evbusiness/chargeready/charging-infra-rebate (2023).

18. Pacific Gas and Electric Company. Empower EV Program. https://www.pge.com/en_US/residential/solar-and-vehicles/options/clean-vehicles/electric/empower-ev-program.page (2023).
19. California Air Resources Board. Clean Cars 4 All. https://ww2.arb.ca.gov/our-work/programs/clean-cars-4-all (2015).
20. Cook County Government. Electric Vehicle Charging Stations Program. *Cook County* https://www.cookcountyil.gov/EVcharging (2023).
21. Mayor's Office of Climate & Environmental Justice. PlaNYC: Getting Sustainability Done. *NYC Mayor's Office of Climate and Environmental Justice* https://climate.cityofnewyork.us/initiatives/planyc-getting-sustainability-done/ (2022).
22. Dallas Climate Action, City of Dallas. Comprehensive Environmental and Climate Action Plan. *Dallas Climate* https://www.dallasclimateaction.com (2020).
23. City of Seattle. Seattle City Light. https://www.seattle.gov/city-light (1910).
24. Agency of Commerce and Community Development. Electric Vehicle Supply Equipment (EVSE) Grant Program. https://accd.vermont.gov/community-development/funding-incentives/electric-vehicle-supply-equipment-evse-grant-program (2014).
25. Southern California Edison. Turn-Key Installation Program. https://www.sce.com/evbusiness/chargeready/turn-key-installation (2022).
26. Department of Environmental Protection. It Pay$ to Plug In. *Drive Green NJ* https://dep.nj.gov/drivegreen/it-pays-to-plug-in/ (2023).
27. New Jersey Department of Environmental Protection. Electric Vehicle and EV Charging Station Grant Program. *Drive Green NJ* https://dep.nj.gov/drivegreen/emobility-eligible-projects/ (2023).

28. Forth. Federal Charging Support. https://forthmobility.org/Federal-Charging-Support (2023).

29. Veloz. Electric For All. *Electric For All* https://www.electricforall.org/ (2018).

30. Petticrew, M. & Roberts, H. *Systematic Reviews in the Social Sciences: A Practical Guide*. (John Wiley & Sons, 2008).

31. Roy, A. & Law, M. Examining spatial disparities in electric vehicle charging station placements using machine learning. *Sustainable Cities and Society* **83**, 103978 (2022).

32. Hsu, C.-W. & Fingerman, K. Public electric vehicle charger access disparities across race and income in California. *Transport Policy* **100**, 59–67 (2021).

33. Li, G., Luo, T. & Song, Y. Spatial equity analysis of urban public services for electric vehicle charging—Implications of Chinese cities. *Sustainable Cities and Society* **76**, 103519 (2022).

34. Cheng, X. & Kontou, E. Estimating the electric vehicle charging demand of multi-unit dwelling residents in the United States. *Environ. Res.: Infrastruct. Sustain.* **3**, 025012 (2023).

35. Carlton, G. J. & Sultana, S. Electric vehicle charging station accessibility and land use clustering: A case study of the Chicago region. *Journal of Urban Mobility* **2**, 100019 (2022).

36. Khan, H. A. U., Price, S., Avraam, C. & Dvorkin, Y. Inequitable access to EV charging infrastructure. *The Electricity Journal* **35**, 107096 (2022).

37. Nazari-Heris, M., Loni, A., Asadi, S. & Mohammadi-ivatloo, B. Toward social equity access and mobile charging stations for electric vehicles: A case study in Los Angeles. *Applied Energy* **311**, 118704 (2022).

38. Min, Y. & Lee, H. W. Social Equity of Clean Energy Policies in Electric-Vehicle Charging Infrastructure Systems. in *Construction Research Congress 2020* 221–229 (American Society of Civil Engineers, Tempe, Arizona, 2020). doi:10.1061/9780784482858.025.

39. Badiei, Y. & Do Prado, J. C. Advancing Rural Electrification through Community-Based EV Charging Stations: Opportunities and Challenges. in *2023 IEEE Rural Electric Power Conference (REPC)* 69–73 (IEEE, Cleveland, OH, USA, 2023). doi:10.1109/REPC49397.2023.00020.
40. Kempton, W., Pearre, N. S., Guensler, R. & Elango, V. V. Influence of Battery Energy, Charging Power, and Charging Locations upon EVs' Ability to Meet Trip Needs. *Energies* **16**, 2104 (2023).
41. Loni, A. & Asadi, S. Data-driven equitable placement for electric vehicle charging stations: Case study San Francisco. *Energy* **282**, 128796 (2023).
42. Rabinowitz, A. I., Smart, J. G., Coburn, T. C. & Bradley, T. H. Assessment of Factors in the Reduction of BEV Operational Inconvenience. *IEEE Access* **11**, 30486–30497 (2023).
43. Asekomeh, A., Gershon, O. & Azubuike, S. I. Optimally Clocking the Low Carbon Energy Mile to Achieve the Sustainable Development Goals: Evidence from Dundee's Electric Vehicle Strategy. *Energies* **14**, 842 (2021).
44. Min, Y., Lee, H. W. & Hurvitz, P. M. Clean energy justice: Different adoption characteristics of underserved communities in rooftop solar and electric vehicle chargers in Seattle. *Energy Research & Social Science* **96**, 102931 (2023).
45. Tsukiji, T., Zhang, N., Jiang, Q., He, B. Y. & Ma, J. A Multifaceted Equity Metric System for Transportation Electrification. *IEEE Open J. Intell. Transp. Syst.* **4**, 690–707 (2023).
46. Dall-Orsoletta, A., Ferreira, P. & Gilson Dranka, G. Low-carbon technologies and just energy transition: Prospects for electric vehicles. *Energy Conversion and Management: X* **16**, 100271 (2022).

47. Desai, J., Mathew, J. K., Li, H. & Bullock, D. M. Using Connected Vehicle Data for Assessing Electric Vehicle Charging Infrastructure Usage and Investment Opportunities. (2022).

48. APA. Racial and ethnic identity. *https://apastyle.apa.org* https://apastyle.apa.org/style-grammar-guidelines/bias-free-language/racial-ethnic-minorities (2024).
