# Supplementary material for: Income and racial disparity in household publicly available electric vehicle infrastructure accessibility
Source: Nat Commun. 2024 Jun 14;15:5106. doi: 10.1038/s41467-024-49481-w (PMC11178907; doi:10.1038/s41467-024-49481-w)
Supplement: Supplementary file 3 — Reporting Summary [file 41467_2024_49481_MOESM3_ESM.pdf]

nature portfolio

Corresponding author(s): Jiehong Lou, Xingchi Shen

Last updated by author(s): Mar 1, 2024

Reporting Summary

Nature Portfolio wishes to improve the reproducibility of the work that we publish. This form provides structure for consistency and transparency in reporting. For further information on Nature Portfolio policies, see our Editorial Policies and the Editorial Policy Checklist.

Statistics

For all statistical analyses, confirm that the following items are present in the figure legend, table legend, main text, or Methods section.

n/a	Confirmed	
☐	☒	The exact sample size (*n*) for each experimental group/condition, given as a discrete number and unit of measurement
☒	☐	A statement on whether measurements were taken from distinct samples or whether the same sample was measured repeatedly
☐	☒	The statistical test(s) used AND whether they are one- or two-sided<br>*Only common tests should be described solely by name; describe more complex techniques in the Methods section.*
☒	☐	A description of all covariates tested
☒	☐	A description of any assumptions or corrections, such as tests of normality and adjustment for multiple comparisons
☐	☒	A full description of the statistical parameters including central tendency (e.g. means) or other basic estimates (e.g. regression coefficient) AND variation (e.g. standard deviation) or associated estimates of uncertainty (e.g. confidence intervals)
☐	☒	For null hypothesis testing, the test statistic (e.g. *F*, *t*, *r*) with confidence intervals, effect sizes, degrees of freedom and *P* value noted<br>*Give P values as exact values whenever suitable.*
☒	☐	For Bayesian analysis, information on the choice of priors and Markov chain Monte Carlo settings
☒	☐	For hierarchical and complex designs, identification of the appropriate level for tests and full reporting of outcomes
☒	☐	Estimates of effect sizes (e.g. Cohen's *d*, Pearson's *r*), indicating how they were calculated

Our web collection on statistics for biologists contains articles on many of the points above.

Software and code

Policy information about availability of computer code

Data collection	There is no software used for collecting the data. We obtained individual-level demographics data from Data Axle. We utilize the publicly available Electric Vehicle Charging Station Locations dataset from Alternative Fuel Data Centre at the US Department of Energy
Data analysis	All data and models are processed in R, Python and Stata 17. The figures are produced in Python, R, and QGIS. All the relevant codes are available on the GitHub from: https://github.com/Jiehonglou/Income-and-Racial-Disparity-in-Household-Publicly-Available-EV-Infrastructure-Accessibility

For manuscripts utilizing custom algorithms or software that are central to the research but not yet described in published literature, software must be made available to editors and reviewers. We strongly encourage code deposition in a community repository (e.g. GitHub). See the Nature Portfolio guidelines for submitting code & software for further information.

Data

Policy information about availability of data

All manuscripts must include a data availability statement. This statement should provide the following information, where applicable:

- Accession codes, unique identifiers, or web links for publicly available datasets
- A description of any restrictions on data availability
- For clinical datasets or third party data, please ensure that the statement adheres to our policy

This paper employs household-level demographic data sourced from Data Axle under a confidentiality agreement that prohibits the sharing of raw data. All the

other data used for this analysis is available from publicly available sources cited or from the authors upon reasonable request. Source data for figures in main text are provided with this paper. All the sharable data are available on the GitHub from:https://github.com/Jiehonglou/Income-and-Racial-Disparity-in-Household-Publicly-Available-EV-Infrastructure-Accessibility

Research involving human participants, their data, or biological material

Policy information about studies with human participants or human data. See also policy information about sex, gender (identity/presentation), and sexual orientation and race, ethnicity and racism.

Reporting on sex and gender	N/A
Reporting on race, ethnicity, or other socially relevant groupings	N/A
Population characteristics	N/A
Recruitment	N/A
Ethics oversight	N/A

Note that full information on the approval of the study protocol must also be provided in the manuscript.

Field-specific reporting

Please select the one below that is the best fit for your research. If you are not sure, read the appropriate sections before making your selection.

☐ Life sciences ☒ Behavioural & social sciences ☐ Ecological, evolutionary & environmental sciences

For a reference copy of the document with all sections, see nature.com/documents/nr-reporting-summary-flat.pdf

Behavioural & social sciences study design

All studies must disclose on these points even when the disclosure is negative.

Study description	We implemented four research designs to investigate the income and racial disparity on accessibility of EV public infrastructure by using a quantitative method
Research sample	Our data sample is 120,962,661 Individual households in the United States, where 7.7% of households are from Urban, and 92.3% from rural. 78% of households are belong to Single Family Dwelling, and 22% belong to Multi-Family Dwelling. The average household income is $78332. There are 72.29% of white households.
Sampling strategy	We obtained individual-level demographics data from Data Axle. We use all the observations, which means we do not use any sampling strategy in this paper. To prevent double counting issues, we include only households indicating their current addresses as their primary addresses. This resulted in 120,962,661 observations, accounting for approximately 97.5% of households in the United States. We then focused our study on Black and White households, resulting in 94,878,711 observations, which accounts for 77% of the households in the United States.
Data collection	We obtained individual-level demographics data from Data Axle. We utilize the publicly available Electric Vehicle Charging Station Locations dataset from Alternative Fuel Data Centre at the US Department of Energy. We started collected the charging station on Dec 29 2022, and finished on the same date, which is also Dec 29 2022.
Timing	The EV charging station data was up to the end of 2022, this is when we finished our analysis. Data Axle is based on 2021 data, since this is the year we have the complete information.
Data exclusions	We exclude all the households who indicted that current address is not their primary addresses.
Non-participation	No Participant is dropped out
Randomization	Our research involved all the households, and the research design did not require randomization. Also randomization is not relevant to our study, because we did not evaluate a policy impact.

Reporting for specific materials, systems and methods

We require information from authors about some types of materials, experimental systems and methods used in many studies. Here, indicate whether each material, system or method listed is relevant to your study. If you are not sure if a list item applies to your research, read the appropriate section before selecting a response.

Materials & experimental systems

n/a	Involved in the study
☒	☐ Antibodies
☒	☐ Eukaryotic cell lines
☒	☐ Palaeontology and archaeology
☒	☐ Animals and other organisms
☒	☐ Clinical data
☒	☐ Dual use research of concern
☒	☐ Plants

Methods

n/a	Involved in the study
☒	☐ ChIP-seq
☒	☐ Flow cytometry
☒	☐ MRI-based neuroimaging

Plants

Seed stocks	*Report on the source of all seed stocks or other plant material used. If applicable, state the seed stock centre and catalogue number. If plant specimens were collected from the field, describe the collection location, date and sampling procedures.*
Novel plant genotypes	*Describe the methods by which all novel plant genotypes were produced. This includes those generated by transgenic approaches, gene editing, chemical/radiation-based mutagenesis and hybridization. For transgenic lines, describe the transformation method, the number of independent lines analyzed and the generation upon which experiments were performed. For gene-edited lines, describe the editor used, the endogenous sequence targeted for editing, the targeting guide RNA sequence (if applicable) and how the editor was applied.*
Authentication	*Describe any authentication procedures for each seed stock used or novel genotype generated. Describe any experiments used to assess the effect of a mutation and, where applicable, how potential secondary effects (e.g. second site T-DNA insertions, mosiacism, off-target gene editing) were examined.*
